# Rapid acquisition of ionomic and morphological data from plant seeds through fast X-ray fluorescence microscopy and computer vision

**DOI:** 10.1016/j.plaphe.2025.100138

**Published:** 2025-11-13

**Authors:** Yu-Peng Zhu, Brigid A. McKenna, Sina Fischer, Peter M. Kopittke, David E. Salt, Xin-Yuang Huang, Fang-Jie Zhao, Zhong Tang, Peng Wang

**Affiliations:** a Nanjing Agricultural University, State Key Laboratory of Crop Genetics and Germplasm Enhancement, College of Resources and Environmental Sciences, Nanjing, Jiangsu, 210095, China; b The University of Queensland, School of Agriculture and Food Sciences, St. Lucia, Queensland, 4072, Australia; c School of Biosciences, University of Nottingham, Sutton Bonington Campus, Loughborough, Leicestershire, LE12 5RD, UK; d Center for Agriculture and Health, Academy for Advanced Interdisciplinary Studies, Nanjing Agricultural University, Nanjing, 210095, China

**Keywords:** μ-XRF, Computer vision, *A. thaliana*, Seeds, GWAS

## Abstract

Plant seeds are one of the most important food sources for humans. As a result, seed morphology and the concentrations of essential and toxic elements in seeds have important implications not only for seed yield and quality, but also for human health. To identify natural variation in the accumulation of various elements in seeds and in seed morphology, high-throughput phenotyping methods are needed. Here, we employed X-ray fluorescence microscopy (μ-XRF) as a method for rapid and high-throughput phenotyping of seed libraries and developed a computer vision-based algorithmic workflow to automatically the extraction of elemental and morphological data from single seeds. This workflow enables rapid segmentation of individual seeds from a genome-wide association study (GWAS) panel with 1163 *A. thaliana* accessions, and facilitates the extraction of elemental and morphological traits at the individual seed level from the μ-XRF image. A total of 7 and 10 loci, respectively associated with the morphology and elemental concentration of *A. thaliana* seeds, were identified. The high-throughput and nondestructive method for automated phenotyping of plant seed libraries developed in this study provides a tool for investigating natural genetic variation controlling the seed mineral accumulation and seed morphogenesis.

## 1 Introduction

Seeds signify the beginning of plant life, providing vital nutrients for germination and initial seedling growth. For humans, seeds also serve as a pivotal resource, not only because they are a fundamental food source but also because they are required for crop production itself [1,2]. Although plants and humans differ in regard to their nutritional requirements, both require adequate amounts of their essential mineral nutrients in order to sustain healthy growth. Mineral elements in the soil are absorbed by plant roots and subsequently transported to various plant stems, leaves and finally to seeds through xylem or phloem transportation pathways. In addition to essential mineral nutrients, seeds may also accumulate toxic elements, such as cadmium (Cd) and arsenic (As), especially where soils are contaminated, or soil conditions favor the mobilization of these elements. Excessive accumulation of toxic elements in seeds poses a threat to human health since they contribute to contamination of food produce [3,4]. Therefore, understanding the mechanisms that regulate the levels of essential and nonessential elements in seeds is crucial not only for studying plant development but also for improving human nutrition and health. This understanding is also the cornerstone of the molecular breeding of crops to biofortify essential mineral elements while concurrently limiting the accumulation of toxic elements [5].

With the advancement of sequencing technologies, genome-wide association studies (GWAS), as an efficient gene mapping technique, have emerged as a pivotal tool for unraveling the genetic basis of complex traits in plants [6,7]. The model plant *A. thaliana* has become an ideal material for GWAS analysis due to its characteristics of continuous self-fertilization, wide distribution, significant genetic variation, and reproducible phenotype [[8], [9], [10]]. Atwell et al. [11] used 107 phenotypes from 191 *A. thaliana* accessions for GWAS analysis and discovered many important alleles, demonstrating the feasibility of GWAS for *A. thaliana* natural population. By integrating high-throughput imaging techniques, Deja-Muylle et al. [12] conducted a GWAS analysis on 17 root architecture traits across 241 Arabidopsis accessions, identifying multiple genes strongly associated with lateral root (LR) development and auxin signaling pathways. Meyer et al. [13] employed a non-invasive time-lapse imaging approach to evaluate early vegetative growth in 382 Arabidopsis accessions, revealing 238 marker-trait associations (MTAs). Furthermore, GWAS coupled with linkage mapping has been utilized to pinpoint genes governing heavy metal accumulation in Arabidopsis leaves [14]. Collectively, these studies demonstrate that GWAS plays a pivotal role in deciphering complex genetic traits and unraveling their regulatory mechanisms.

The exploration of genes governing plant elemental uptake, translocation, and accumulation through forward genetics, and elucidating their molecular mechanisms through functional genomics, relies on both precise plant phenotype data and genomic data. However, the technological development of approaches for acquiring high-quality phenotypic data through high-throughput methods lags behind the generation of high-throughput genomic data. The lack of large-scale phenotype analysis platforms has now become a major bottleneck constraining the advancement of crop functional genomics, greatly limiting the progress of genomics-assisted crop improvement programs [[15], [16], [17]].

The primary methods for determining mineral elements include molecular spectroscopy, atomic spectroscopy, and mass spectrometry [[18], [19], [20]]. Inductively coupled plasma mass spectrometry (ICP-MS) is widely used to identify variant seeds with altered concentrations for a range of elements [[21], [22], [23], [24], [25]]. For example, Yang et al. [26] determined the concentrations of 15 mineral elements in rice grain from a diverse panel of 529 accessions using ICP-MS and identified candidate genes for 42 loci related to elements content in rice grain through GWAS. Although ICP-MS is effective in analyzing bulk elemental concentrations in plant organs, it is a time-consuming and labor-intensive process that involves destructive analysis and necessitates extensive sample preparation, such as grinding and acid digestion, particularly when studying large numbers of samples [20]. X-ray fluorescence microscopy (μ-XRF) is a high-throughput, non-destructive elemental imaging technique that can be used for quantifying mineral elements in plant seeds [[27], [28], [29], [30], [31]]. By using synchrotron-based μ-XRF as a phenotyping tool for examining elemental distribution in rice (*Oryza sativa*) seeds, Ren et al.[32] identified a total of 692 putative mutants and 65 loci associated with the spatial distribution of elements from a M1 rice mutant library and 533 diverse rice accessions, respectively. Although ImageJ based semi-automate method was applied to extract elemental data from the μ-XRF images [29], it still requires experienced human operation, resulting in limited element data extraction efficiency and possible operator error. Therefore, the development of a fully-automated method to extract seed phenotype data from μ-XRF images will greatly expand the availability of μ-XRF based techniques for large-scale seed mineral element determination.

Image-based phenotyping has emerged as a critical tool, empowering researchers to explore diverse plant traits at various spatiotemporal and spectral resolutions. It has been suggested that effective image-based plant phenotyping requires a combined approach in the fields of image processing for feature extraction and machine learning for data analysis [33]. Various combined computer vision (CV) and machine learning (ML) analytical methods have been devised to enable the automation of organ-level phenotypic analysis, including leaves, roots, and reproductive organs [[34], [35], [36], [37], [38]]. However, several challenges remain when trying to automate organ-level phenotyping using computer vision and machine learning techniques. First, plant organs such as leaves, roots, and reproductive organs vary widely in their shapes, sizes, textures, and colors. This variability can make it challenging to develop models that can accurately detect and analyze these organs across different species and environmental conditions [[39], [40], [41]]. Second, in image scenarios, the region of interest is often surrounded by complex backgrounds, occlusions, or overlapping structures, thereby making it difficult for computer vision algorithms to accurately segment and analyze the target region [40,42,43]. Third, advanced imaging technologies such as synchrotron radiation typically generate high-dimensional data, including a large amount of spatial and spectral information, and processing these complex datasets requires efficient algorithms and techniques to extract useful information and features [44,45].

To address these challenges, we have developed an original algorithm flow that combines μ-XRF imaging and computer image analysis to perform single seed segmentation, external morphology extraction, and element trait characterization from nearly 12,000 seeds of 1163 *A. thaliana* accessions. This method can quantify the concentrations of 15 mineral elements and seven morphological features from μ-XRF images at the single seed scale. By combining the phenomics and genetic data, we identified multiple genetic loci related to seed element concentration and morphology through GWAS analysis. This μ-XRF image-based methodology can also be applied to phenotyping seeds of major field crops.

## 2 Materials and methods

### 2.1 Plant materials and μ-XRF analyses

**Fig. 1:**
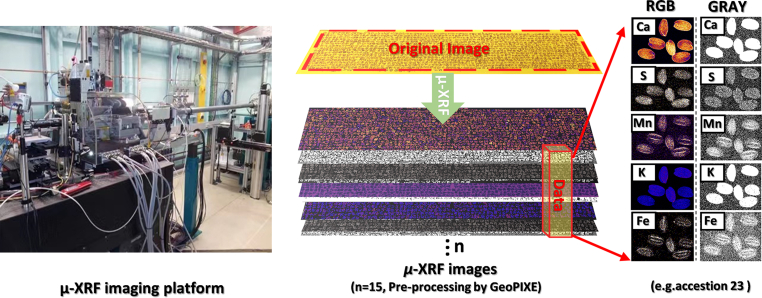
μ-XRF imaging workflow. Schematic of the μ-XRF imaging platform (left) and *A. thaliana* seed elemental image (right).

With the development of second-generation sequencing technologies, the “1001 Genomes” project [10,46], led by the Max Planck Institute for Developmental Biology in Europe, has established a data-sharing platform (www.1001genomes.org), which includes information on thousands of different ecotypes of *A. thaliana* worldwide. This platform contains whole-genome sequencing data for 1135 accessions of *A. thaliana*, methylation data for 1028 *A. thaliana* transcriptome data for 998 *A. thaliana* and geographic location information for all of them, which provided the genotype data basis for this research, In this study, we used 1163 *A. thaliana* germplasm resources collected from different ecological niches around the world (Fig. S1A, Data S1), which were planted in Aberdeen (UK) and harvested under the same conditions [22], We used the μ-XRF microimaging platform(Fig. 1) to analyze the concentration of mineral elements in the seeds of 1163 *A. thaliana* accessions. We taped 5–20 seeds of each accession on the tape (1 ​mm ​× ​1 ​mm Kapton Tape, Alexnld) (Fig. S1B) with manual marking and each film was then sandwiched between two layers of 4 ​μm thick xylene films (Spex® SamplePrep XRF window film, USA) using Super glue (US company Super Glue Corporation,USA) (Fig. S1B). Seeds were analyzed at the X-ray fluorescence microscopy (XFM) beamline at the Australian Synchrotron, Melbourne, Australia [[47], [48], [49]]. The XFM beamline utilizes an in-vacuum reducer to produce a brilliant X-ray with a Si (111) monochromator and Kirkpatrick-Baez mirrors to deliver a monochromatic focused beam to the specimen [50]. The X-ray fluorescence emitted from the sample was recorded by the 384-element Maia detector placed in a backscatter geometry [51]. The incident beam, with an energy of 18.6 ​KeV, is capable of detecting elements ranging from sulfur (S) to strontium (Sr) at the K-edge with a total photon flux of 5.5 ​× ​10^8^ photons per second. The scanning process, covering 158.58 million pixels over an area of 119,000 ​× ​33,200 ​μm required approximately 35 ​h. The data cube of the acquired XRF spectral images was saved in .*dai* file format, with the total file size of the data cube being approximately 20.5 gigabytes (GB). All XRF spectra were preprocessed using GeoPIXE (v7.4) and images were generated using dynamic analysis method [52]. This process generated color images with relative quantification of red, green, and blue (RGB) (Fig. S2). Additionally, spectral images encompassing distinct bands were stored in grayscale maps with absolute quantification (Fig. S3). Each image boasts a resolution of 23,881 ​× ​6641 pixels. GeoPIXE can automatically subtract background and analyze overlapping peaks and relate the detected X-ray signal in each pixel to the amount of fluorescent X-rays calculated based on the assumed *A. thaliana* tissue composition and the thickness of the sample, thus, semi-quantitative values can be calculated for all the different elements [53].

### 2.2 Developing algorithms for extracting seed elemental and morphological phenotypes

We developed an automated algorithm using the Python programming language to rapidly extract the seed phenotypes from μ-XRF images, which includes 15 elemental phenotypes (As, Ca, Cl, Cr, Cu, Fe, K, Mn, Ni, P, Rb, S, Se, Sr and Zn) (Fig. S4) and seven morphological phenotypes (length, width, perimeter, area, aspect ratio, eccentricity and roundness) (Fig. S5). In order to obtain these phenotypes, we saved relative quantitative images (RGB) and absolute quantitative images (GRAY) of the various elements with GeoPIXE. Our algorithms integrated various image processing and vector analysis algorithms, including threshold segmentation [54], supervised machine learning [[55], [56], [57], [58]], digital morphology [59,60], watershed segmentation [61], and Harris corner detection [62], the specific processes are as follows. Firstly, we obtained the individual accession of regions through digital morphology, and for areas that cannot be obtained, we set up a mouse *callback* function to listen to the mouse click event to obtain the coordinate information and used *line_aa* function in scikit-image library to connect the coordinates and split the adherent regions to obtain the image mask 1 of individual varieties of regions. Secondly, we sorted the coordinates of the center of mass of the accession region from top to bottom and from left to right based on the numbering algorithm. Thirdly, we use supervised machine learning algorithms to obtain image masks for single seeds. For the adhesive seed regions, we developed an automated segmentation process using watershed and Harris corner detection algorithms to segment the adhesive seed regions (i.e. identify the ROI corresponding to each individual seed) and generate image mask 2. Finally, we obtained the average elemental concentration and morphological data of each individual seed by overlaying two masks (mask1 and mask2) with the absolute quantitative grayscale image generated by GeoPIXE through automated trait extraction.

### 2.3 Hardware and software foundations for algorithm construction

The algorithm development process was based on the Python (v3.10.9) programming language. The main open-source scientific development libraries used in this study include the image processing libraries scikit-image (v0.19.3) and opencv-python (v4.7.0), data visualization libraries Matplotlib (v3.7.0) and Seaborn (v0.12.2), data computation libraries SciPy (v1.10.0) and NumPy (v1.23.5), machine learning library scikit-learn (v1.2.1), and data analysis library Pandas (v1.5.3). The development process described above was conducted utilizing the Jupyter Notebook as the integrated development environment (IDE) for coding, testing, and execution on a personal computer equipped with an Intel® Core™ i9-12900H CPU @ 2.60 ​GHz, 48.0 ​GB DDR5 memory, 1 ​TB hard drive, and an NVIDIA GeForce GTX 3060 dedicated graphics card running the Windows 11 operating system.

### 2.4 Manual data acquisition

In this study, elemental concentrations of seeds were determined using ICP-MS to validate the results obtained from μ-XRF imaging. Seeds were digested with concentrated nitric acid using a microwave oven digestor (CEM, Mars, USA), and each digestion batch of ablated samples included duplicates of a blank control and a standard reference material (GSB-23a) to ensure the precision of the analytical procedure. The concentrations of elements in the digested solution were determined using ICP-MS (PERKIN-ELMER, NexION 300X, USA) (Data S4). The seed morphological data, including perimeter, area, and length versus width of each seed, were manually measured from a total of 1367 seeds of 100 accessions using ImageJ (v1.52a), based on color difference images (RGB).

### 2.5 GWAS analysis

All GWAS analyses in this study were conducted on the GWA-Portal website (https://gwas.gmi.oeaw.ac.at/) [63]. The seed elemental and morphological traits of *A. thaliana* seeds obtained from the μ-XRF platform, were used for GWAS to identify gene loci associated with these phenotypes. The entire analysis employed the Accelerated Mixed Model (AMM) [64] association analysis method. Finally, the physical map of the identified loci was visualized using the MapGene2Chrom (MG2C) online platform (http://mg2c.iask.in/mg2c_v2.1/)[65].

### 2.6 Statistical analysis

The experiment adopts a random block design, and 5 to 10 seeds are selected from each genotype for statistical analysis. All data are expressed as mean ​± ​standard deviation. The statistical analysis program used is Python 3.10.9 and R 4.4.1. An analysis of variance (ANOVA) was used to test the significance, followed by comparisons of treatment means using Tukey's test (*P* ​< ​0.05). Fig. 6, Fig. S4 and Fig. S5 were visualized using Matplotlib [66], Fig. 7 and Fig. S8 were visualized using ggplot2 3.5.1[67].

## 3 Results

### 3.1 Trait analysis workflow based on single seed

**Fig. 2:**
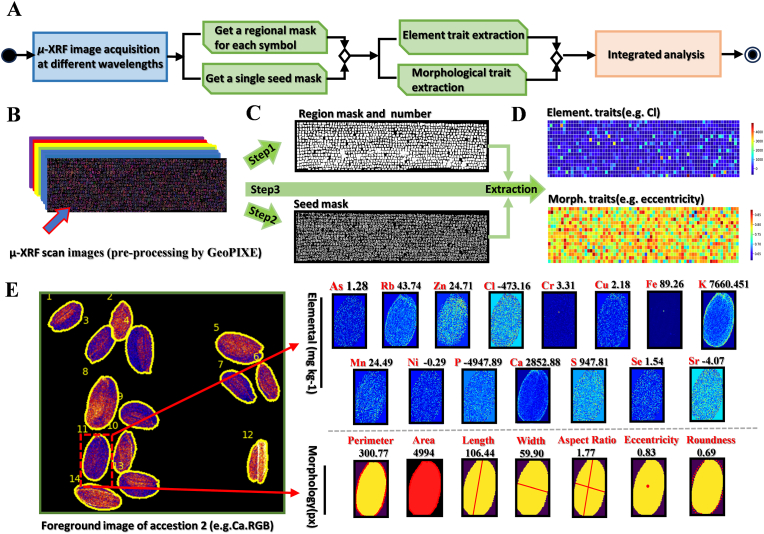
Integrated workflow for seed morphological and elemental phenotyping. (A) Image processing pipeline for automated seed segmentation and trait extraction. (B) Elemental imaging via μ-XRF coupled with GeoPIXE (v7.2) spectral pre-processing. (C) Image processing and machine learning based segmentation outputs: accession number-associated mask (up) and individual seed masks (down). (D) Trait extraction (Accession number-associated regions are expressed as mean values and overlaid with pseudo-color). (E) 17 elemental traits (As, Ca, Cl, Cr, Cu, Fe, K, Mn, Ni, P, Rb, S, Se, Sr, and Zn) and 7 morphological traits (perimeter, area, length, width, aspect ratio, eccentricity, and roundness) based on a single seed (e.g.Ca.accession.seed 11, all description of the traits are listed in Table 1).

After acquiring the μ-XRF image data, we developed a workflow for seed phenotypic trait analysis (Fig. 2A). Initially, utilizing the data cube derived from the μ-XRF platform, we generated both a color image (RGB) and a grayscale image (GRAY) that preserve the quantitative fidelity of spectral information of each element through the dynamic analysis performed using GeoPIXE software (Fig. 2B). Subsequently, image processing pipelines were implemented to generate masks for distinct accession regions and individual seed regions (Fig. 2C), Finally, an automated extraction of image traits based on the accession region numbers obtained from the sorting algorithm was used to complete the quantitative analysis of single seed morphology and elemental composition (Fig. 2D). Morphological traits obtained from single seed mask included seed area, length, width, perimeter, aspect ratio, roundness and eccentricity (Fig. 2E). The formula for eccentricity is: $E=\sqrt{1-\frac{L^{2}}{w^{2}}}$ (1), where E is the eccentricity of the seed, L and W are the lengths of the long and short axes of the ellipse, respectively. The formula for roundness is $R=\frac{4\pi \times A}{P^{2}}$  (2), where R is the roundness of the seed and A and P are the area and perimeter of the ellipse, respectively. The elemental traits that can be characterized for the average spectral reflectance in the selected 15 bands include As, Ca, Cl, Cr, Cu, Fe, K, Mn, Ni, P, Rb, S, Se, Sr, and Zn (Fig. 2E). (Due to the limited sensitivity of μ-XRF technology for detecting low atomic number elements (such as Mg), and the fact that some elements (such as Mo) have concentrations lower than the instrument's detection limit in the target sample, these elements were not included in the quantitative analysis range in this study. So, ICP-MS technology was used to supplement the determination of relevant elements [29]. Following morphological and elemental analyses, extracted trait data were stored in a CSV file (Data S2, Data S3), with detailed descriptions provided in Table 1, providing a quantitative basis for further analysis. Under a standard computational configuration (Intel® Core™ i9-12900H CPU @ 2.60 ​GHz, 48 ​GB DDR5 memory, 1 ​TB SSD, and NVIDIA GeForce RTX 3060 GPU), the complete automated pipeline processed a single ultra–high-resolution μ-XRF image (23,881 ​× ​6641 pixels, ∼158 million total pixels) containing approximately 11,285 seeds from 1163 accessions within 18–19 ​h. This end-to-end process included raw image pre-processing, segmentation, and the extraction of both elemental data (15 elements) and morphological traits (7 per seed).

**Table 1:** All traits and descriptions obtained based on the μ-XRF platform.

Traits type	Image trait description
Morphological traits	1. Maximum length of ​*A. thaliana* seeds (*Length*, *computer vision indicators*)
	2. Maximum width of ​*A. thaliana* seeds (*Width, computer vision indicators)*
	3.Projected area of ​*A. thaliana* seed area (*Area, computer vision indicators*)
	4. Projected perimeter of the seed region of ​*A. thaliana* (*Perimeter, computer vision indicators*)
	5. Measure the deviation of an elliptical target relative to a circle, quantifying information about the shape of the target (*Eccentricity, computer vision indicators*)
	6. A common metric used to quantify how closely the shape of an object in an image resembles a circle is circularity (*Roundness, computer vision indicators*)
	7. Ratio of maximum length to width of seeds, an important indicator used to quantify morphology (*Aspect Ratio, computer vision indicators*)
Elemental traits	8. Major elements (P and K); medium elements (Ca and S); minor element (As、Cl、Cr、Cu、Fe、Mn、Ni、Rb、Se、Sr and Zn) (u-XRF)

All trait algorithms integrated in jupyter notebook of python.

### 3.2 Segmentation and sorting of accession regions

**Fig. 3:**
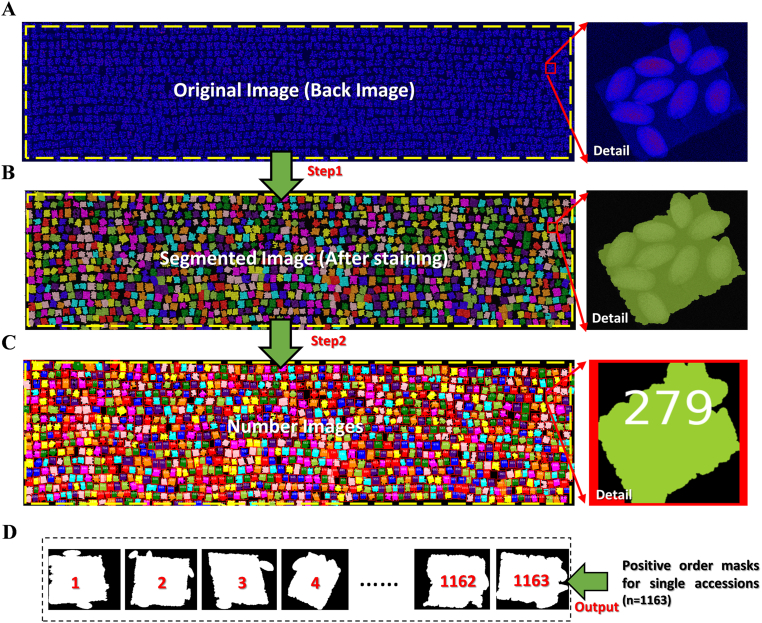
Segmentation and numbering of 1163 A. thaliana accession number-associated regions. (A) μ-XRF background image generated via GeoPIXE dynamic analysis. (B) Segmenting accession number-associated regions and overlaid with pseudo-color. (C) Numbering of accession number-associated regions. (D) Positive order masks for single accession number-associated regions.

We used the background image (RGB), which includes complete tape regions, obtained from the μ-XRF platform as the original image for processing (Fig. 3A). In the RGB (red, green, blue) color space, both the tape and seed regions were distinctly visible as bright blue. After converting the image from the RGB to the Lab (CIE L∗a∗b∗) space, the lightness (L∗) channel for the tape region was close to the maximum value, while the *a*∗ and *b*∗ channels were close to the mid-range value. A binary image of the tape region was obtained through the minimum cross-entropy threshold [68], which minimizes cross-entropy. To fill in the holes in the accession region mask, the numerical morphology closure operation was utilized. The unfilled parts were filled using the *binary_fill_holes* function from the SciPy function package. For the sticking and burring parts at the edges of the region in the preliminary segmentation, we performed subdivision and smoothing through the open operation to acquire more refined label regions. However, the unsegmented areas were manually defined using the *SetMouseCallback* function from the opencv library in conjunction with the *line_aa* function from the scikit-image library. After obtaining the segmentation lines, we used the *logical_xor* function from the Numpy library in combination with the initial segmentation mask to obtain the final image mask (Fig. 3B).

To distinguish between different accessions and to correlate the obtained trait data with each accession, it is necessary to assign numerical identifiers to the regions. Given the high resolution of the-images and the irregular distribution of regions per accession, existing algorithms struggled to assign accurate numerical labels to these regions. Therefore, we developed a new numbering method utilizing the *line_aa* function from the scikit-image library to delineate row regions within the image mask. A total of 17 row regions were marked and then filled using the *flood_fill* function from scikit-image to produce the masks for these 17 regions. These masks were combined with the final mask using the *logical_and* function from the Numpy library to extract masks corresponding to all accessions within the 17 regions. At the same time, we obtained the center-of-mass coordinates of each accession, and sorted the accessions from left to right based on their order within the 17 regions. The sorting of centroid points matched the accession regions in the image as shown in Fig. 3C, with the corresponding actual accession numbers displayed in white. Due to the abundance of accession regions, we employed the *label* function from the scikit-image library to establish a one-to-one correspondence between the labeled accession regions and the accurately sorted coordinates of center-of-mass points. This facilitated the generation of individual masks for each accession region (Fig. 3D), which can be preserved for further use.

### 3.3 Seed mask acquisition based on machine learning

**Fig. 4:**
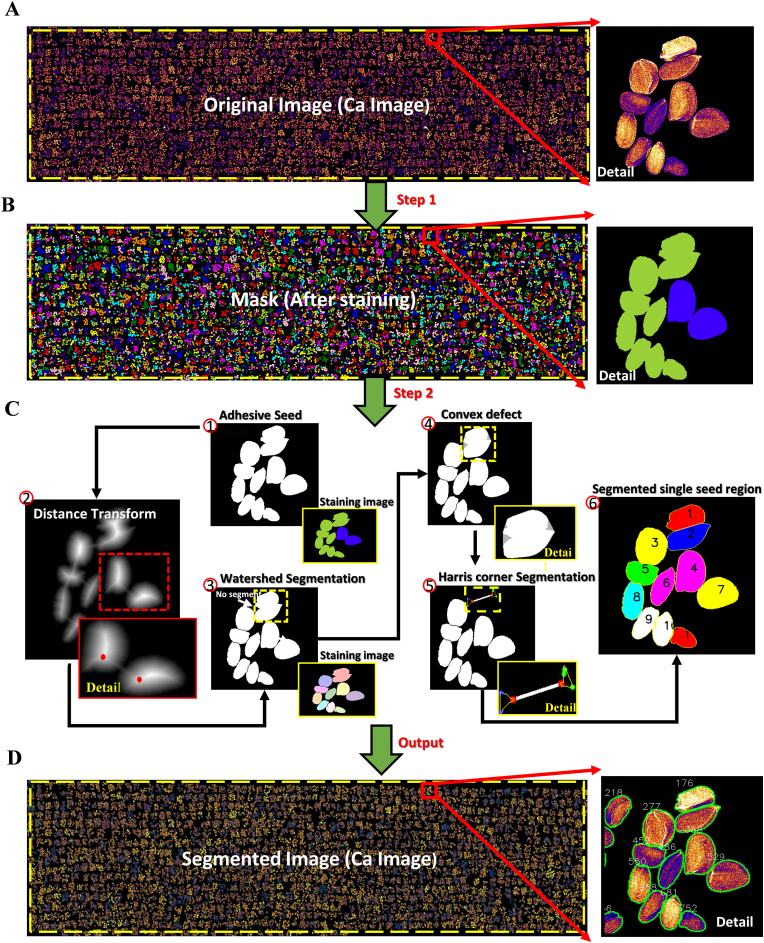
Seed mask acquisition and attached seed segmentation based on machine learning and image processing algorithms. (A) Calcium (Ca) color images generated via GeoPIXE dynamic analysis. (B) Seed mask acquisition based on machine learning and overlaid with pseudo-color. (C) Algorithmic segmentation process for adhered seeds. (D) Segmented single seed and cover the contour line.

Given that a clear contrast existed between the individual seed regions and the background, the Ca elemental image obtained from the μ-XRF platform served as the original processed image (Fig. 4A). The seed mask acquisition was transformed into a binary classification problem, encompassing pixels from both seed and non-seed regions. These pixels, based on two color spaces, namely RGB and Hue-Saturation-Value (HSV), with a total of six channels, constituted the dataset (six feature values, 4416 records) for machine learning. The dataset was divided into a training set (n ​= ​3532) and test set (n ​= ​884) in a ratio of 8:2. Four supervised machine learning algorithms, K-Nearest Neighbor (KNN) [58], Support Vector Machine (SVM) [57], Random Forest (RF) [55], and Extreme Gradient Boosting (XGBoost) [56] were used for training. We evaluated the classification models using a confusion matrix [69] (Fig. S6), Accuracy, Precision, Recall, and F1-Score [70](Table S1). Ultimately, the optimal model, XGBoost, was selected to perform image segmentation for separating seeds from the background (Fig. 4B).

### 3.4 Adherent seed segmentation algorithm

**Fig. 5:**
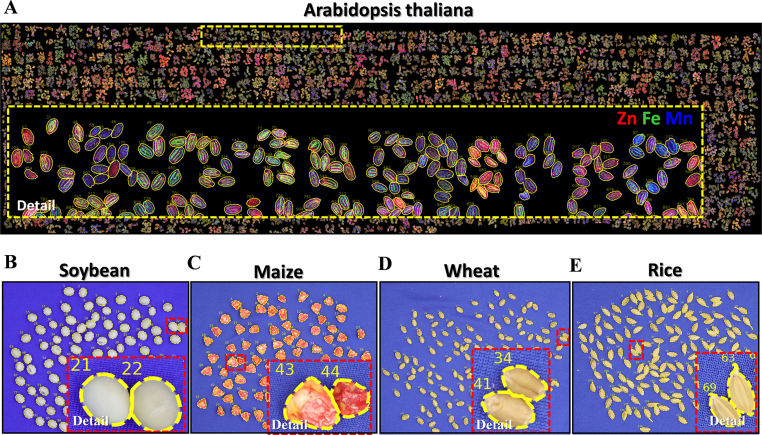
Cross-species validation of segmentation algorithms in major crops. Representative segmentation results of (A) Arabidopsis thaliana, (B) soybean, (C) maize, (D) wheat and (E) rice, demonstrating the generalizability of the machine learning-based segmentation pipeline. Yellow division dashed line highlight precision in delineating seed/plant architectures under varying morphological complexities.

**Fig. 6:**
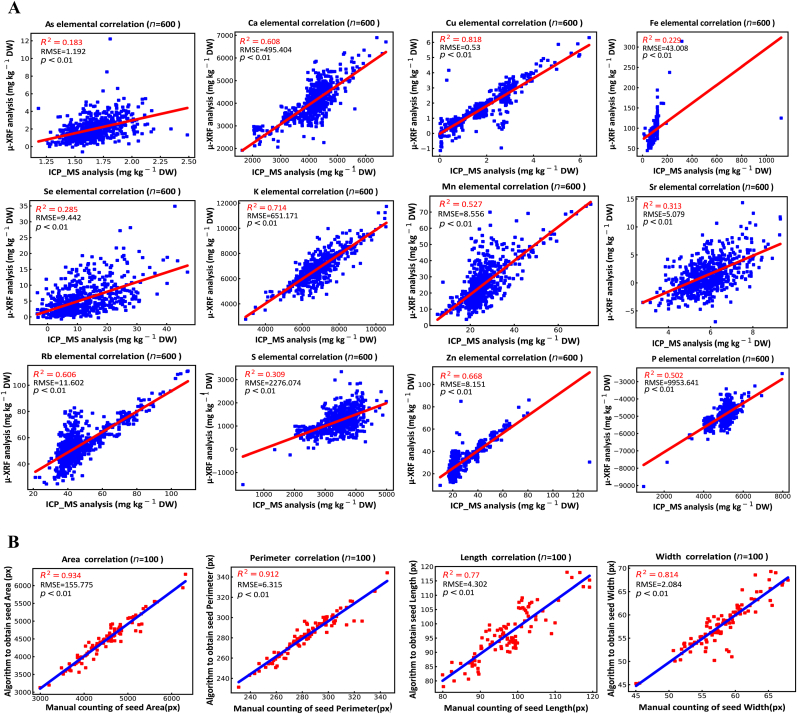
Validation of automated phenotyping through Pearson correlation analysis. (A) Correlation between μ-XRF/algorithm-derived elemental concentrations and ICP-MS measurements. (B) Correlation between algorithm-generated morphological traits and ImageJ-based manual measurements.

Target extraction of plant phenotypic images often encounters a challenging problem in processing adherent regions (Figs. 4C–1). Although digital morphology processing has addressed the issue of the irrelevant regions in the image and the missing problem inside the samples, it struggles with the effective processing of the adherent samples [71,72]. In the present study, we proposed an automated segmentation process as shown in Fig. 4C. Segmentation of seeds with irregular shapes and overlapping was achieved using the watershed algorithm [61] based on a mathematical topology approach (Figs. 4C–2), and by setting the splitting parameter to 25 based on a preferential determination of parameters. To further enhance the segmentation quality, we implemented a post-processing filtration step to address occasional over-segmentation by the watershed algorithm. Specifically, we calculated the median area of all segmented objects and established a threshold at 0.75 times this median value. Objects with areas below this threshold were considered potential over-segmentation artifacts and were systematically filtered out, thereby ensuring the high accuracy and reliability of the final phenotypic dataset. For areas that remained unsegmented (Figs. 4C–3, the yellow dashed), we first measured the area of all objects in the seed mask. Then, using 1.25 times the median area as the seed area threshold, we filtered out individual seed masks and identified regions with more than one seed (Figs. 4C–3, the yellow dashed). Where these regions had multiple seeds overlaying each other, we used the convex packet method (the *convex_hull_image* function from the scikit-image library) to aggregate the overlying seeds into a larger convex packet (Fig. 4C–4, the yellow dashed). Subsequently, the convex defect regions and their contours were identified using the *logical_xor* function from the Numpy library. The Harris corner detection algorithm [62] was then employed to detect all inflection points along the contour of each convex defect region. These points were marked in red based on their euclidean distance from the nearest corner point (Figs. 4C–5, the yellow dashed). Connecting these corner points using the *line_aa* function from the scikit-image library resulted in the segmentation of the seed area. This iterative process continued until all remaining seed areas were smaller than the predefined seed area threshold (Figs. 4C–6, Figs. 4D and 5A). It should be noted that in rare instances, segmentation via the convex defect approach may still produce over-segmented results. To address this limitation, we applied the same area based filtration strategy used for watershed post-processing to eliminate these artifacts.

**Fig. 7:**
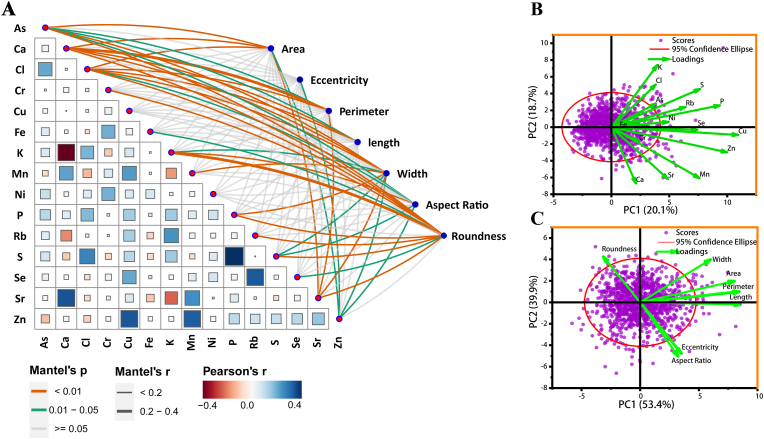
Multidimensional trait relationship analysis. (A) Mantel test evaluating associations between elemental and morphological traits. Edge width represents Mantel's r values (distance correlations), while edge color denotes Pearson correlation coefficients (color bar) and statistical significance (*P*-values derived from 9999 permutations; dashed lines: *P* ​< ​0.05). (B) Principal Component Analysis (PCA) of seed morphological traits. (C) PCA of seed elemental composition profiles.

To assess the suitability of the algorithm across different plant species, we systematically tested it on seed images (n ​= ​20) with varying densities from four distinct crop species: rice, wheat, maize, and soybean (The image processing workflow is shown in Fig. S7 (e.g. rice)). The performance was rigorously assessed using two standard metrics, the Intersection over Union (IoU) and the Dice coefficient, which measure the pixel-wise agreement between the algorithm's output and manually annotated ground truth masks. Our method achieved high mean IoU scores of 81.85 ​± ​3.18 (rice), 87.22 ​± ​2.19 (wheat), 86.96 ​± ​2.86 (maize), and 86.42 ​± ​1.77 (soybean), with corresponding Dice coefficients of 89.86 ​± ​1.96, 93.12 ​± ​1.28, 92.94 ​± ​1.68, and 92.65 ​± ​1.03, respectively. These consistent and high scores across all four species (as detailed in Table 2) quantitatively demonstrate that the algorithm can accurately and robustly segment both non-adherent and adherent seeds (Fig. 5B–E), confirming its broad applicability for diverse crop plants.

**Table 2:** Segmentation performance on 20 seed images exhibiting varying population densities.

	Soybean	Maize	Wheat	Rice
IoU (%)	86.42 ​± ​1.77	86.96 ​± ​2.86	87.22 ​± ​2.19	81.85 ​± ​3.18
Dice coefficient (%)	92.65 ​± ​1.03	92.94 ​± ​1.68	93.12 ​± ​1.28	89.86 ​± ​1.96

The Intersection over Union (IoU) measures the overlap between the predicted segmentation and the ground-truth mask, calculated as the area of intersection divided by the area of union. The Dice coefficient quantifies the similarity between two sets, computed as twice the area of overlap divided by the total number of pixels in both sets. Both metrics range from 0 % (no overlap) to 100 % (perfect alignment), with higher values indicating better segmentation accuracy.

### 3.5 Comparison of μ-XRF and ICP-MS for elemental concentrations

We measured the concentrations of several elements (K, Mn, As, Cu, Ca, Se, Rb, and Sr) (Data S4) in whole seeds for 600 *A. thaliana* accessions using ICP-MS and compared these results with the corresponding elemental concentrations automatically extracted from μ-XRF images using the method developed above (Fig. 6A). A significant relationship (*P* ​< ​0.01) was observed between concentrations measured by μ-XRF and ICP-MS across all elements examined, with the R^2^ values ranging from 0.18 to 0.82. Elements such as Ca, Cu, K Rb and Zn exhibited relatively high correlations (R^2^ ​= ​0.50–0.82), indicating that robust detection performance of μ-XRF for these elements. These elements often exist as soluble ions or are associated with relatively uniformly distributed proteins, show more consistent distribution across various seed tissues. Consequently, the intensity signals from the μ-XRF scan are more representative of the average content in the whole seed, leading to higher correlation. In contrast, As, Fe, Se and S showed weaker correlations (R^2^ ​= ​0.18–0.31), which can be largely attributed to both technical and biological factors. From a technical perspective, μ-XRF imaging has inherent limitations related to penetration depth, which depends on the atomic number of each element. Heavier elements such as Rb and Sr can be detected throughout the entire seed (approximately 2–3 ​mm), whereas lighter elements such as P and S are primarily detected in superficial layers (10–50 ​μm) [29]. Biologically, the heterogeneous distribution of certain elements further contributes to the observed discrepancies. For instance, As and Se tend to accumulate in specific seed tissues such as the seed coat or embryo as part of detoxification or storage strategies [[100], [101], [102]]. When the μ-XRF scans crosses such localized “hotspots”, the resulting signals may not linearly reflect total elemental concentrations quantified by ICP-MS, thereby reducing the overall correlation. Similarly, sulfur in seeds exists in multiple chemical forms, including inorganic sulfate, S-rich storage proteins (e.g. in gliadins), and glutathione [103,104]. As μ-XRF detects total S irrespective of speciation, spatial heterogeneity in these compounds can also lead to divergence from bulk ICP-MS measurements. Collectively, differences in sampling volume, detection depth, and chemical sensitivity explain the weak correlations observed for As, Se, and S. In addition, we measured the morphological traits including area, perimeter, length, and width of the seeds for 100 accessions by using ImageJ software to compare with the traits obtained through the algorithm we devised. A highly significant positive correlation was observed between the morphological traits quantified by the two methods, with the R^2^ values of 0.93, 0.91, 0.77, and 0.81 for area, perimeter, grain length, and grain width, respectively (Fig. 6B). These results suggest that the morphological traits derived from the μ-XRF image through the algorithm in this study are robust and reliable.

### 3.6 Mantel test analysis for elemental and morphological traits

To investigate how elemental concentrations relate to seed morphology, we conducted a Mantel test analysis (Fig. 7A). The analysis revealed that Ca exhibited a significantly positive correlation with Sr, but a negative correlation with K and Rb, aligning with prior studies [22]. This pattern can be attributed to their shared and divergent transport pathways: Ca and Sr, being chemical analogs, are likely transported non-selectively by the same families of transporters, such as the Ca^2+^ permeable channels (e.g. *GLRs, CNGCs*) and Ca^2+^/H^+^ exchangers (*CAXs*) [105]. In contrast, the negative correlation between Ca and K/Rb reflects their antagonistic roles in cellular signaling and ion homeostasis, as well as competition for binding sites in the cell wall, such as Ca cross-links pectin while K^+^ influences osmotic potential and cell expansion [105,106]. A strong association was also observed among Zn, Cu, and Mn, suggesting coordinated regulation by metal transporters with broad substrate specificity, such as the *ZIP* (ZRT/IRT-like Protein) and *NRAMP* (Natural Resistance-Associated Macrophage Protein) families, which are known to mediate the uptake and translocation of these divalent cations in plants [107,108]. Regarding associations with morphological traits, we observed a significant correlation between Ca and seed perimeter, likely reflecting Ca's role in cell wall strengthening and cell division, and between K and seed roundness, which may be related to K's function in regulating osmotic pressure and cell shape during seed development (Fig. 7A). These results imply that the accumulation of these elements in seed may influence morphological seed development.

### 3.7 Principal component analysis

To investigate the relationships among seed elemental concentrations and morphological traits and to identify the underlying factors driving trait variation, principal component analysis (PCA) was performed for all the seed elemental concentrations and morphological traits obtained from μ-XRF image through the algorithm developed in the present study (Fig. 7B and C). For the results of seed elements, PC1 explained 20.1 ​% of the trait variance with Cu, Zn, P showing high positive loadings. These results suggested that accessions with high PC1 scores had high Cu, Zn, and P concentrations in the seed, and vice versa. PC2 explained 18.7 ​% of the total variance, and the loading on PC2 was high for K, Cl, S, suggesting that PC2 is representative of these elements. The PCA also supported the clustering of the different elements found in the elemental association analysis. For example, PCA plots typically clustered together chemical analogs such as Ca and Sr, as well as elements like Mn, Zn, and Cu, which can be attributed to the relatively broad selectivity of certain uptake and transport proteins for these elements, such as the aforementioned *ZIP* and *NRAMP* families for Mn, Zn, and Cu, and the non-selective cation channels for Ca and Sr [[108], [109], [110]] (Fig. 7B). Notably, the co-localization of S with K and Cl in the PCA may reflect their shared role in osmotic and charge balance, as well as the fact that S is primarily transported as sulfate via sulfate transporters, whose expression can be co-regulated with K^+^ and Cl^−^ channels during seed filling[111,112]. For the results of seed morphological traits, PC1 accounted for 53.4 ​% of the variation, with the highest contribution from the traits of area, perimeter and length. PC2 accounted for 39.9 ​% of the variation with the highest contribution from the traits of roundness and width. The first two principal components together explained 93.3 ​% of the total variation, indicating that these two principal components effectively capture the overall information and can be used as quantitative indices to characterize seed morphological traits (Fig. 7C).

Based on the 95 ​% confidence intervals (t-distribution, α ​= ​0.05), 57 accessions (Data S5) exhibited extreme ionomic phenotypes for at least one of the 15 elements, while 69 accessions (Data S6) displayed extreme seed morphology phenotype in one or more of seven measured traits (Fig. 7B and C).

### 3.8 GWAS analysis of μ-XRF imaging-based seed elemental and morphological traits

**Fig. 8:**
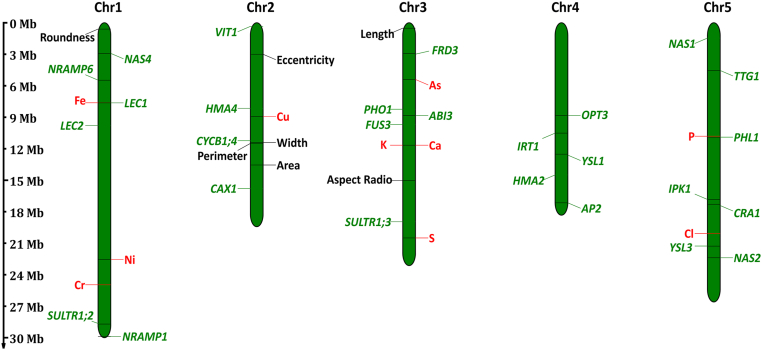
Chromosomal distribution of trait-associated loci and known candidate genes. Physical positions of 17 loci across the five *Arabidopsis thaliana* chromosomes are annotated based on genomic coordinates (scale in megabases, Mb). Loci linked to seed morphological traits are marked in black, elemental accumulation-associated loci in red, and previously reported candidate genes in green (refer to Table 2 for detailed genetic annotations).

GWAS analysis was performed on 1163 natural accessions of *A. thaliana* using data on 15 seed elements content traits seven seed morphological traits obtained from the μ-XRF platform, the results of the GWAS manhattan plots and Q-Q plots are shown in Fig. S8. A total of 17 GWAS loci were obtained (Detailed locus information can be found in Data S7). To identify candidate genes responsible for each locus, we firstly extracted all genes within 200 ​Kb of peak SNPs above the significance threshold (Data S8). By taking into account their annotations, homologous genes functions, and distances from the peak SNPs, we identified 7 significant SNPs linked to seed morphological traits and 10 associated with elemental concentrations. Notably, based on association strength, functional relevance, and physical proximity to the peak SNPs, two highly promising candidate genes were identified. A significant locus on chromosome 5 *(chr05_10953961*, *P* ​= ​7.12 ​× ​10^−7^) was located near *PHL1* (AT5G29000), which encodes a *MYB* transcription factor known to regulate the phosphate starvation response. For seed iron concentration, a significant signal (*chr01_7727677*) was detected within the genomic region of *LEC1* (AT1G21970), a well-characterized regulator of embryogenesis and seed development[85]. These high-confidence candidate loci present strong targets for subsequent mechanistic exploration. The 7 loci linked to seed shape phenotypes (including seed area, length, width, perimeter, eccentricity, roundness and aspect ratio), are distributed on the Chromosome 3 of the *A. thaliana* genome (Fig. 8). The 10 loci linked to seed concentrations of elements including Fe, Ni, Cr, Cu, As, P, K, Ca, S and Cl, of which Fe, Ni and Cr on *Chr1*, Cu on *Chr2*, As, P, K, Ca and Son *Chr3*, and Cl on *Chr5* (Fig. 8). For comparative purposes, we mapped the chromosomal positions of previously reported genes associated with seed elemental accumulation and morphogenesis in *A. thaliana* (Comprehensive gene functional annotations are presented in Table 3), these loci will facilitate further investigations into the genetic architecture and natural variation underlying seed morphogenesis and elemental accumulation in *A. thaliana*.

**Table 3:** Annotations of functional genes that have been reported.

Genes	Accession number	Chr	Coordinates(bp)	Annotation
*NAS1*	AT5G04950	5	1457641–1459039	Participate in the absorption of root iron and its transportation to the aboveground part [73,74]
*NAS2*	AT5G56080	5	22711141–22712441	Catalytic synthesis of nicotinamide, promoting long-distance transportation of iron and seed iron storage [73]
*NAS4*	AT1G09240	1	2984829–2986231	Regulating the distribution of iron in aboveground tissues such as leaves and seeds[73,75]
*VIT1*	AT2G01770	2	334663–336462	Mediate the transport of iron ions to vacuoles and regulate the distribution of iron in seeds[24,76]
*HMA2*	AT4G30110	4	14720062–14725311	Zinc ion transporters regulate the distribution of zinc from roots to aboveground parts and seeds[77,78]
*HMA4*	AT2G19110	2	8279437–8286445	Participate in the transport and accumulation of zinc ions, enhance cadmium tolerance[77,79]
*PHO1*	AT3G23430	3	8387398–8393467	Participate in the transportation of phosphorus from roots to aboveground parts, affecting the phosphorus content of seeds[80]
*PHL1*	AT5G29000	5	11021980–11024865	Encoding a *MYB* transcription factor, which acts redundantly with *PHR1* to regulate responses to P starvation[81,82]
*CAX1*	AT2G38170	2	15989092–15993285	Regulating the storage of calcium ions in vacuoles and affecting seed calcium content[83]
*SULTR1;3*	AT3G51895	3	19251287–19255922	Sulfur transporters regulate the accumulation of sulfur in seeds[84]
*LEC1*	AT1G21970	1	7727577–7729649	Regulating endosperm synthesis and seed maturation[85,86]
*LEC2*	AT1G28300	1	9896867–9899876	Regulating embryonic development, accumulation of storage substances (oils, proteins), and seed maturation[85]
*FUS3*	AT3G26790	3	9853828–9856212	Regulating early embryonic development and seed maturation[85,87]
*ABI3*	AT3G24650	3	8997370–9001185	Regulating seed maturation and dormancy, in synergy with *LEC1*[88]
*TTG1*	AT5G24520	5	8370317–8372847	Synthesis of seed coat pigments (flavonoids) and differentiation of epidermal cells[89]
*CYCB1;4*	AT2G26760	2	11401118–11403433	Cyclins significantly affect the seed size of *Arabidopsis thaliana*[7]
*AP2*	AT4G36920	4	17400724–17403392	Regulating endosperm development and seed size[90]
*IRT1*	AT4G19690	4	10707344–10709015	Root absorption is the primary pathway for iron uptake by plants, indirectly influencing iron concentration in grains[91].
*FRD3*	AT3G08040	3	2566196–2569700	The transport of chelated iron in the xylem affects iron concentration in grains[92].
*YSL1*	AT4G24120	4	12524186–12527347	Phloem allocation directly transports iron and zinc to the developing seeds[93].
*YSL3*	AT5G53550	5	21755291–21758950	Phloem allocation directly transports iron and zinc to the developing seeds[93].
*SULTR1;2*	AT1G78000	1	29329329–29333212	Root uptake is involved in the accumulation of selenium and sulfur in grains[94].
*CRA1*	AT5G35880	5	17756238–17758336	Seed coat storage directly determines the calcium content of the grain[95].
*OPT3*	AT4G16370	4	9247302–9250343	Phloem allocation and signaling regulate the distribution of metals to grains and systemic homeostasis[96].
*IPK1*	AT3G07370	5	17166702–17169459	Embryonic storage determines the storage forms and bioavailability of phosphorus and minerals[97].
*NRAMP1*	AT1G80830	1	30372668–30376065	Root manganese uptake indirectly affects the manganese content in grains [98].
*NRAMP6*	AT1G15960	1	5481665–5486105	The partitioning of iron and manganese within the embryo affects their final distribution [99].。

Accession number according to TAIR (https://www.arabidopsis.org).

## 4 Discussion

μ-XRF is a powerful tool for element characterization as it enables high-throughput and non-destructive analyses of elemental distribution. By utilizing the high brightness, tuneability, and high resolution of monochromatic X-rays at synchrotron facilities, it is possible to study plant tissues and cell structures using approaches not possible previously, thereby providing new information regarding basic biological processes such as plant growth, development, and organ formation [113]. Recent advances in μ-XRF have not only significantly increased sensitivity but also reduced data acquisition times by orders of magnitude, making this technique suitable for high-throughput screening of variation in elemental concentrations in plant seeds [48]. It is important to acknowledge, however, that access to synchrotron-based μ-XRF remains limited by the availability of beamtime and the geographical distribution of facilities, which currently constrains its widespread adoption in routine plant phenotyping. However, recent advances in benchtop μ-XRF technology now allow for sub-50 μm spatial resolution and multi-element mapping capabilities comparable to synchrotron-based setups, making them suitable for routine phenotyping in laboratory environments [114]. The integration of automated sample handling and data analysis pipelines such as the one developed in this study can further improve the efficiency and accessibility of μ-XRF-based phenotyping, regardless of the X-ray source employed. Moreover, coupling our automated image analysis pipeline with these compact systems would enable decentralized, scalable, and cost-effective elemental phenotyping across research institutions. Additionally, we have noted that our algorithmic framework is hardware-agnostic and can be directly adapted to emerging high-throughput seed screening platforms that employ fluorescence, hyperspectral, or micro-CT imaging. This flexibility ensures that the computational framework developed in this study remains applicable even as imaging technologies evolve.

In this regard, Ren et al*.*[32] developed a high-throughput phenotyping method for elemental analysis of rice seeds using μ-XRF combined with an ImageJ analysis pipeline. However, this semiautomated method still demands considerable manual intervention. Particularly when processing complex, high-resolution images, the analysis protocols require case-specific customization to accommodate diverse experimental requirements. In addition, the semiautomated method also requires avoiding adhesion during seed preparation before μ-XRF imaging. Currently, there is no method that effectively combines μ-XRF imaging with advanced computer vision technology to extract seed phenotypic features automatically. To address this gap, we developed a high-throughput phenotyping workflow that minimizes manual input during the initial data preparation stage and enables fully automated, high-quality extraction of both elemental and morphological traits at the single-seed scale from μ-XRF images, providing a comprehensive tool for automated seed phenotyping. In comparative benchmarking, manual analysis using ImageJ requires approximately 30 ​s per seed to measure four basic morphological traits, equating to nearly 100 ​h of continuous work for a dataset of 12,000 seeds. In contrast, our automated workflow achieves the same level of analysis more than 5.5 times faster, while maintaining superior consistency and reproducibility. Additionally, the integrated design of our pipeline allows simultaneous extraction of elemental and morphological data from a single μ-XRF scan, eliminating the need for separate measurement sessions typically required by conventional methods. Leveraging the inherent high-throughput capability of μ-XRF imaging (∼10 ​s per seed), the overall system achieves an estimated ∼1000-fold increase in phenotyping throughput compared with traditional ICP-MS–based elemental analysis. Existing studies have examined seed morphology phenotyping based upon machine vision [[115], [116], [117]]. Most of them focus on the seeds from the same accession, but do not address trait differences between different accessions and individuals within the population. Furthermore, these previously-published methods for the acquisition of single seed traits often face issues such as the difficulties of adhesion segmentation of multiple seeds and incomplete retention of complete edge information [116,118,119], resulting in low efficiency in algorithms for single seed phenotype acquisition. In the present study, we developed a method that incorporates an accession segmentation algorithm, and a machine learning-based seed mask acquisition process, which serves as the foundation for extracting phenotypic traits of individual seeds, taking into account differences between different accessions and individual seeds within the same accession. Additionally, by optimizing the automated image segmentation algorithm, our method has also effectively addressed the issue of seed adhesion. This enables us to accurately obtain the morphological traits of single seeds, thus improving the acquisition of elemental traits. To evaluate the applicability of the algorithm across different scenarios, we conducted tests on seed images of various species, including rice, wheat, maize and soybean (Fig. 5). The results consistently demonstrated effective segmentation, proving our algorithm to be a versatile tool for multiple plant species, and providing a robust seed phenotyping solution for crops.

Currently, over two billion people worldwide suffer from micronutrient deficiencies such as Fe and Zn [120,121]. Biofortification to increase micronutrients in staple foods such as crop seeds is one approach to provide the minimum amount of dietary micronutrients for these populations. One of the major constraints in developing biofortified seeds with high concentrations of essential mineral nutrients is the limited understanding of the mechanisms that regulate mineral nutrient and trace element concentrations in seeds [122,123]. Given that the μ-XRF approach enables high-throughput measurement of seed elemental traits, studies linking the elemental imaging capabilities of μ-XRF with genetic approaches present significant opportunities for the examination of seed elements associated genes in planta [29,124,125,126]. Additionally, seed size is also an important agronomic trait that largely determines yield and is an important focus of research. In the present study, by applying our automated phenotyping pipeline to μ-XRF images, we performed a GWAS in *A. thaliana* seeds, identifying 10 loci associated with seed elemental content and 7 loci linked to seed morphological traits. Candidate genes could be further identified in these loci based on functional annotations and sequence variations. For example, the locus on chromosome 5 (*chr05_10953961*, *P* ​= ​7.12 ​× ​10^−7^) associated with seed phosphorus content is located near *PHL1* (PHOSPHATE STARVATION RESPONSE 1-LIKE 1, AT5G29000), which encodes a *MYB* transcription factor that regulates phosphate starvation responses alongside *PHR1* [81,82,127,128]. This suggests that natural variation at this locus influences *PHL1*-mediated phosphorus allocation during seed development. Additionally, a seed iron-associated signal *(chr01_7727677*) co-localizes with *LEC1* (LEAFY COTYLEDON1, AT1G21970), a central transcriptional regulator in embryogenesis and seed maturation, controlling the accumulation of storage compounds[129,130]. This finding implies a previously unexplored genetic connection between iron homeostasis and seed development. We hypothesize that *LEC1* may indirectly influence seed iron loading by regulating the expression of iron transporters or chelators during seed maturation. Beyond elemental traits, our GWAS also identified significant associations with seed morphological traits. Notably, a locus on chromosome 2 (*chr02_11719904*, *P* ​= ​2.81 ​× ​10^−6^) was strongly associated with both seed perimeter and seed width, two key indicators of seed morphology. A nearby gene, *CYCB1;4* (AT2G26760), has previously been identified through GWAS as a direct regulator of seed size in plants [7]. *CYCB1;4* encodes a cyclin protein that modulates endosperm cell proliferation during seed development, thereby influencing seed size and weight [7]. The significant association detected near this locus not only validates the reliability of our automated phenotyping data but also highlights its relevance to seed morphological variation. Several other candidate genes identified in our study encode transcription factors from the *WRKY* and *bHLH* families. Previous studies have shown that these families play key roles in regulating seed size and weight across plant species: certain *WRKY* proteins integrate hormonal signals to modulate seed development, while specific *bHLH* factors directly control cell proliferation and expansion in developing seeds [[131], [132], [133], [134]]. Nonetheless, further studies are required to validate the functions of these candidate genes.

In summary, we have developed a high-throughput and non-destructive method for automated phenotyping of seed libraries containing genetic variants for individuals with altered elemental concentrations and morphogenesis. Our method can be effectively combined with GWAS to identify genetic loci and candidate genes involved in seed mineral accumulation and morphogenesis. By simultaneously drawing element and morphological locus maps, our research addresses a key gap in seed biology, which is the integration of multi trait associations in GWAS analysis. This highlights the ability of our method to reveal the multiple regulatory factors of seed development. Finally, the candidate genes identified in our study lay the groundwork for further studies to elucidate the molecular mechanisms controlling seed mineral accumulation and seed size in *A. thaliana*.

## Author contributions

P.W., P.M.K., and D.E.S. conceptualized the study and designed the experiments. P.W., B.A.M., S.F., P.M.K. and Y.P.Z. performed the experiments. Y. Z. developed the algorithm and conducted data analysis. Y.P.Z. and P.W. drafted the manuscript. X.Y.H. and F.J.Z. revised the manuscript. All authors contributed to the discussion and the revision of the manuscript.

## Declaration of competing interest

The authors declare that they have no known competing financial interests or personal relationships that could have appeared to influence the work reported in this paper.

## Data Availability

The source code and algorithm of u-XRF are distributed under the MIT License, which permits academic use, distribution, and reproduction subject to the terms of the license (https://opensource.org/license/MIT/), unless otherwise specified. Supporting source code, Web of Science Global Science Publications data, and additional datasets can be accessed at https://github.com/The-Wang-Lab-NAU/u-XRF/for download and upload. Furthermore, source code, data, and user guides are openly available upon reasonable request.
