# Supplementary material for: Rapid acquisition of ionomic and morphological data from plant seeds through fast X-ray fluorescence microscopy and computer vision
Source: Plant Phenomics. 2025 Nov 13;7(4):100138. doi: 10.1016/j.plaphe.2025.100138 (PMC13109303; doi:10.1016/j.plaphe.2025.100138)
Supplement: Multimedia component 1 [file mmc1.pdf]

SUPPLEMENTARY DATA

Rapid acquisition of ionomic and morphological data from plant seeds through fast X-ray fluorescence microscopy and computer vision

Yu-Peng Zhu[1], Brigid A. McKenna[2], Sina Fischer[3], Peter M. Kopittke[2], David E Salt[3], Xin-Yuang Huang[1], Fang-Jie Zhao[1], Zhong Tang[1,*], Peng Wang[1,4*]

[1]*Nanjing Agricultural University, State Key Laboratory of Crop Genetics and Germplasm Enhancement, College of Resources and Environmental Sciences, Nanjing, Jiangsu 210095, China*

[2]*The University of Queensland, School of Agriculture and Food Sciences, St. Lucia, Queensland 4072, Australia*

[3]School of Biosciences, University of Nottingham, Sutton Bonington Campus, Loughborough, Leicestershire LE12 5RD, UK

[4]*Center for Agriculture and Health, Academy for Advanced Interdisciplinary Studies, Nanjing Agricultural University, Nanjing 210095, China*

*Corresponding Author: Peng Wang (p.wang3@naju.edu.cn) and Zhong Tang (tangzhong@njau.edu.cn)

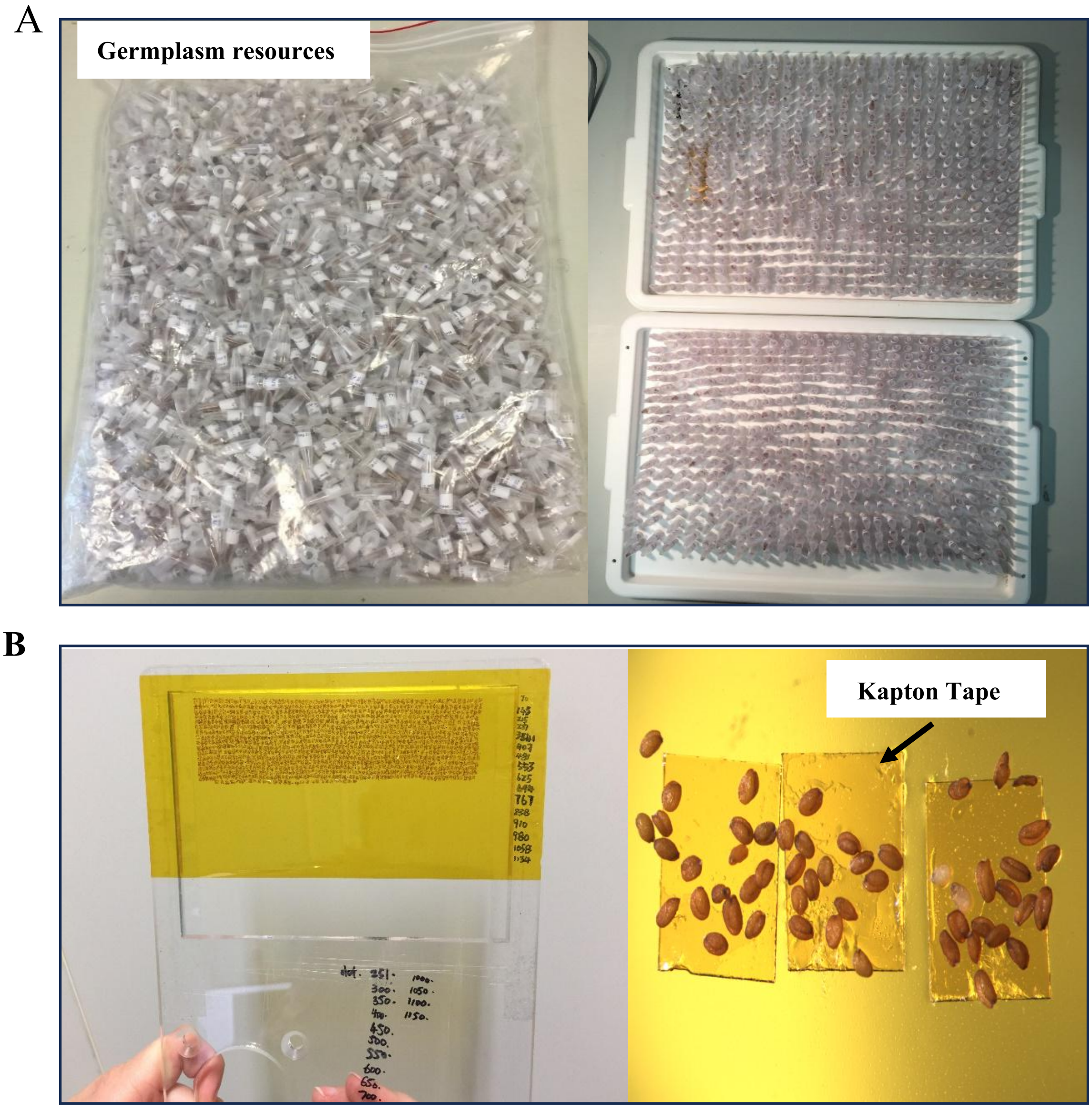

Fig. S1. Sample collection and preparation. (**A**) Collected seeds of 1163 accessions of Arabidopsis thaliana from different ecological niches worldwide. (**B**)1163 accessions in total (including two accessions with no ID on their tubes, and two accessions list in the spreadsheet list but not in the samples)

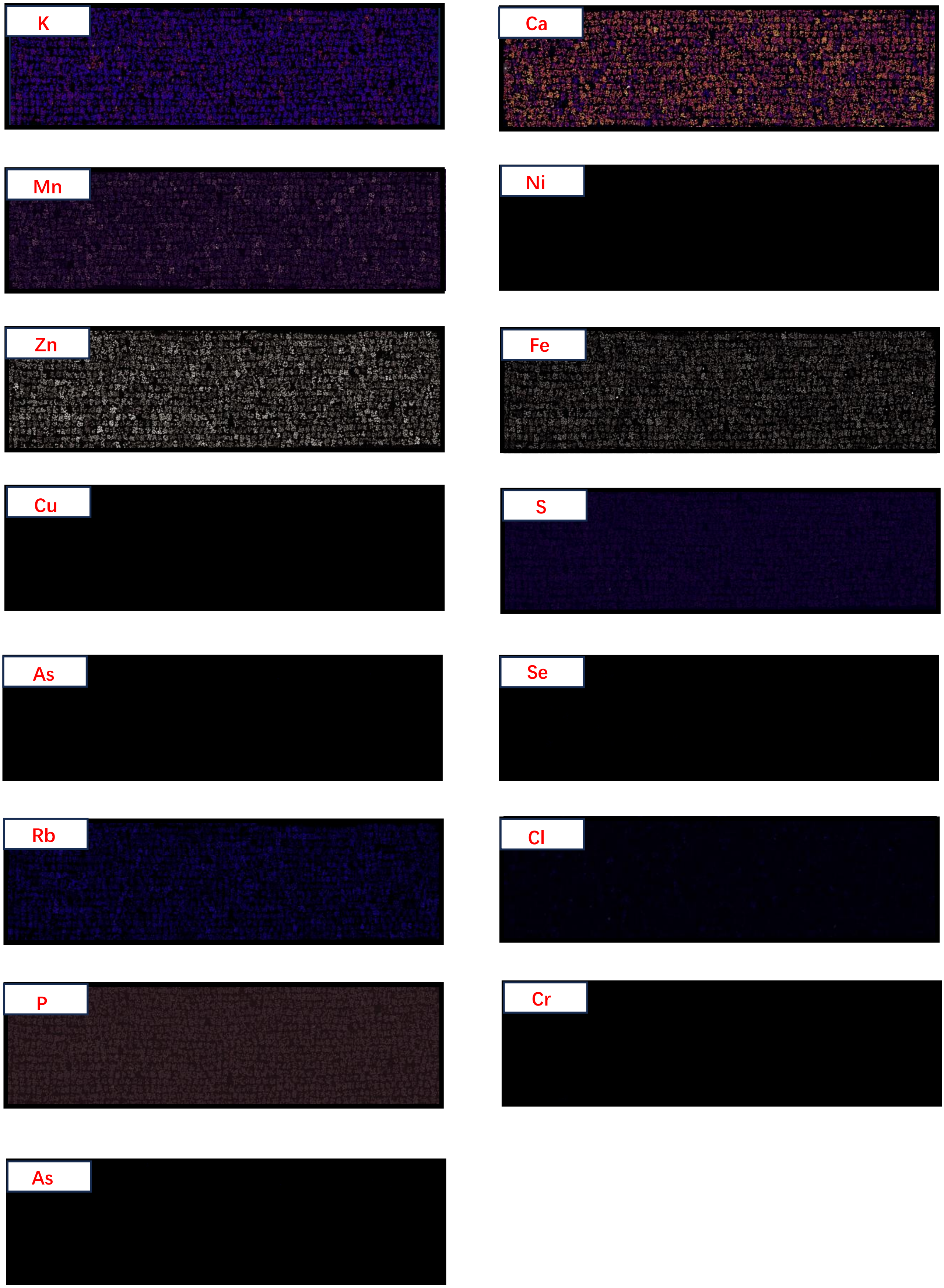

Fig. S2. Generate relative visualization images of elements using dynamic analysis methods (RGB).

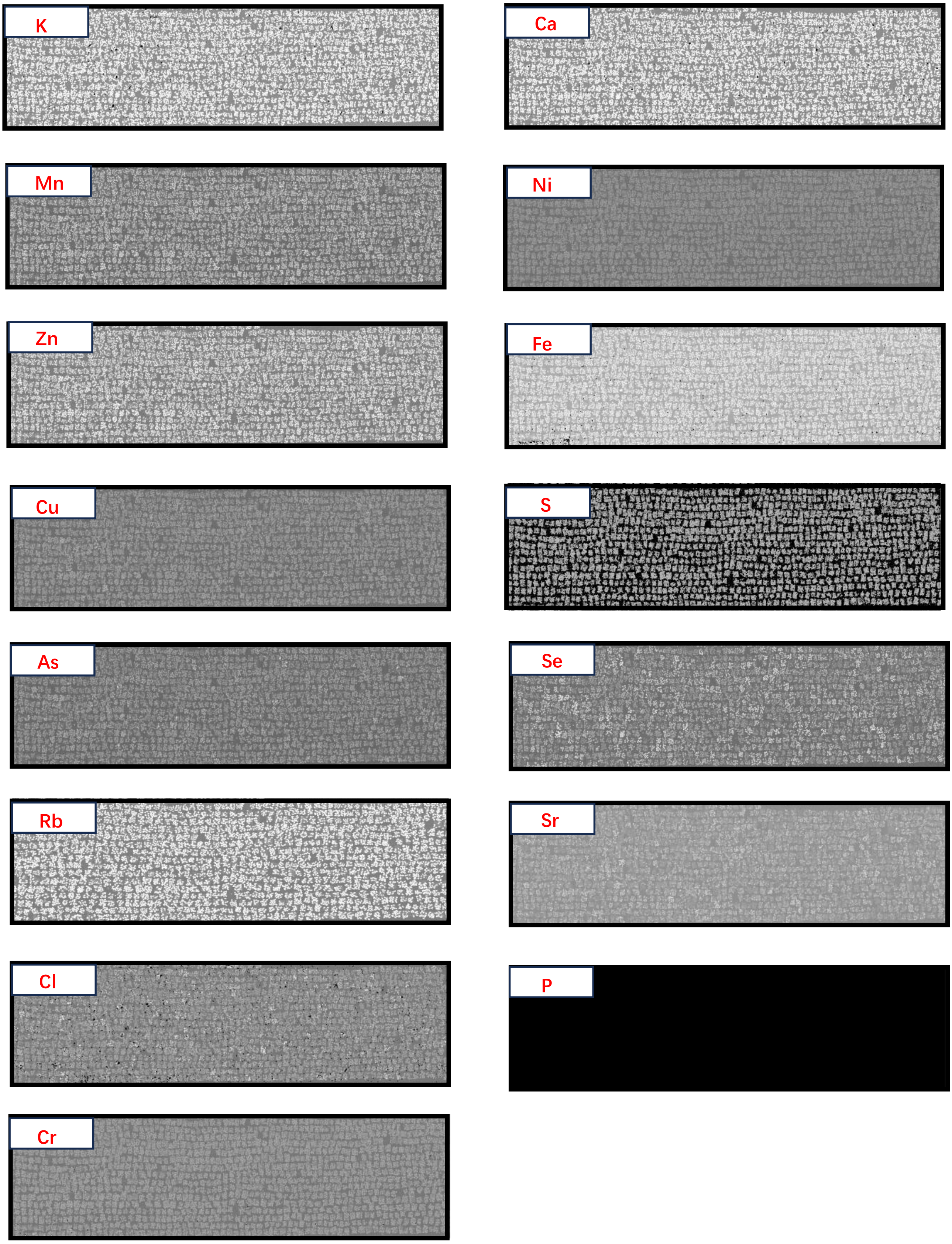

Fig. S3. Generate images with quantitative data elements using dynamic analysis methods, quantitative data is saved in a single-channel grayscale map (GRAY).

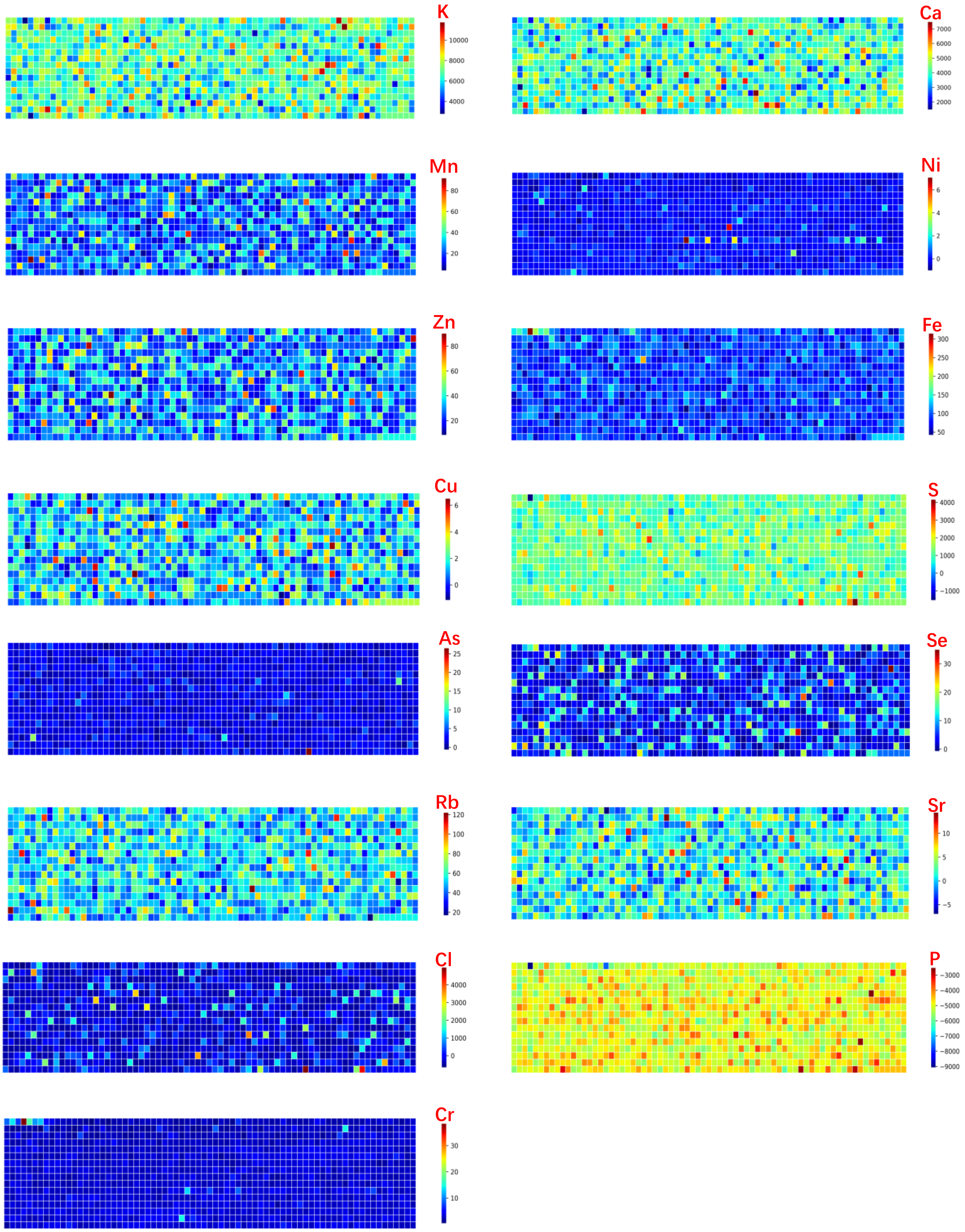

Fig. S4. Pseudo-color element content measured based on u-XRF and image processing algorithms (Mean value of each accession).

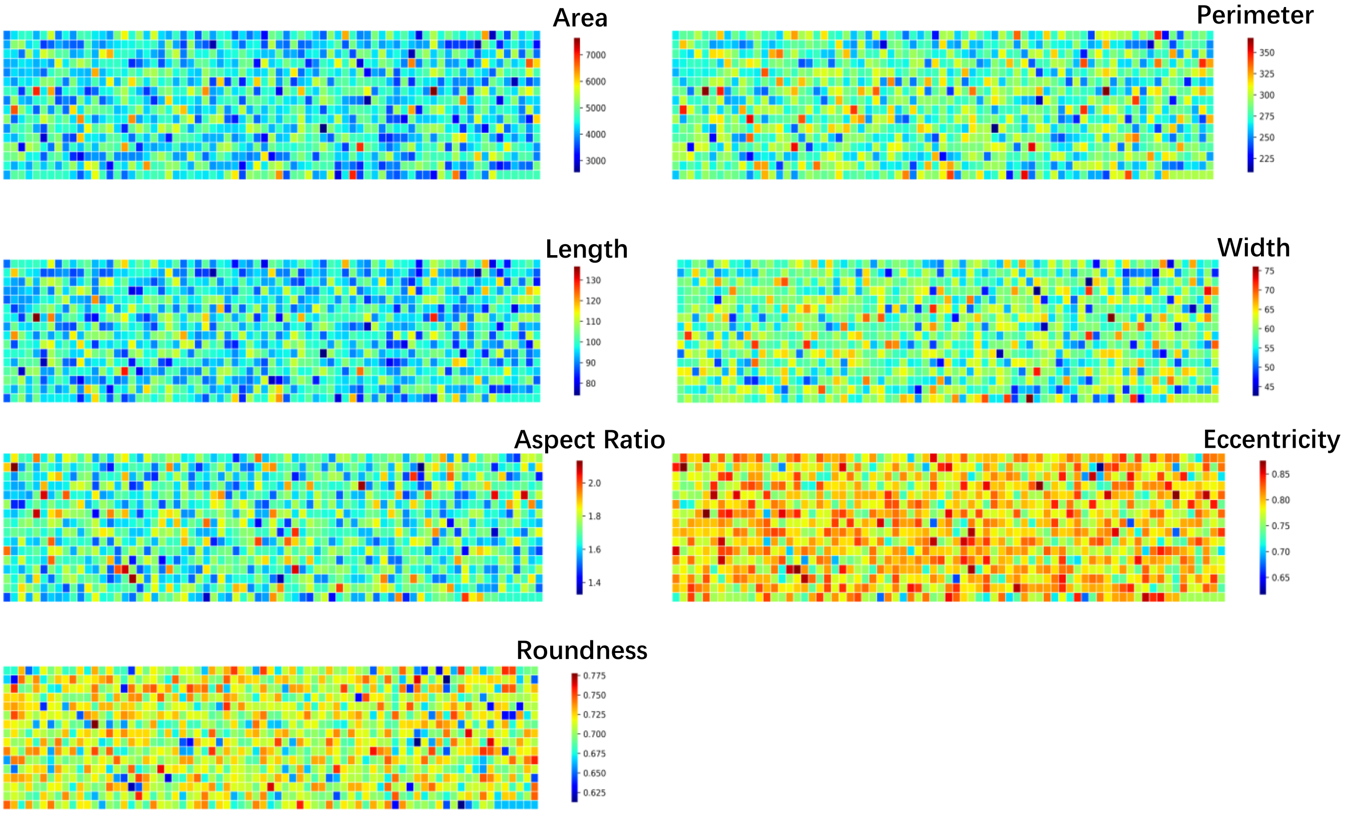

Fig. S5. Pseudo-colored morphological content measured based on u-XRF and image processing algorithms (Mean value of each accession).

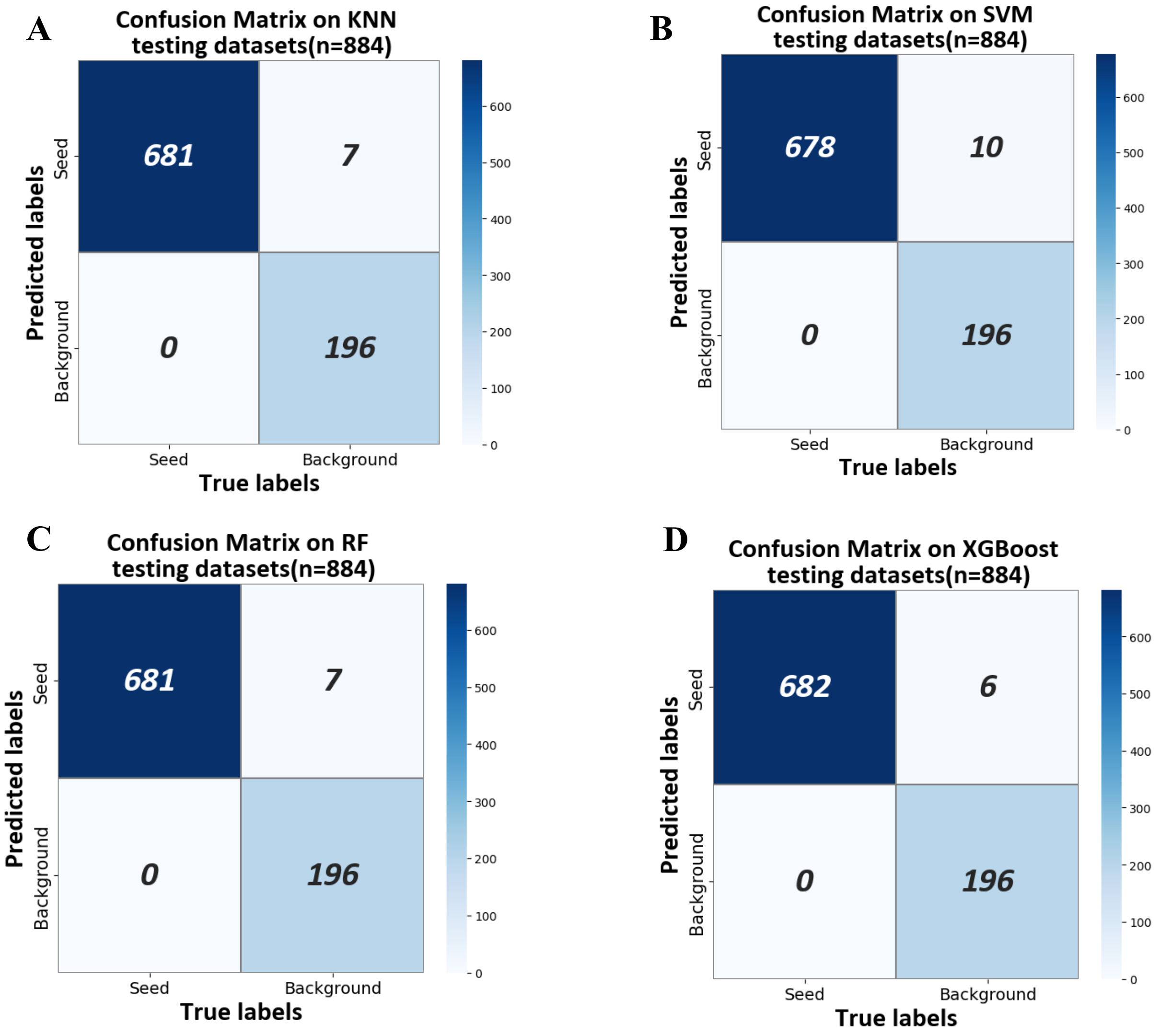

Fig. S6. Classification performance evaluation of four computational models via confusion matrices. Normalized confusion matrices for (**A-D**) Model 1 to Model 4 on the independent test set (n = 884 samples). Rows correspond to ground-truth classes, columns to predicted classes. Color gradients indicate classification frequencies (scale: 0–100%), with diagonal elements representing correct predictions.

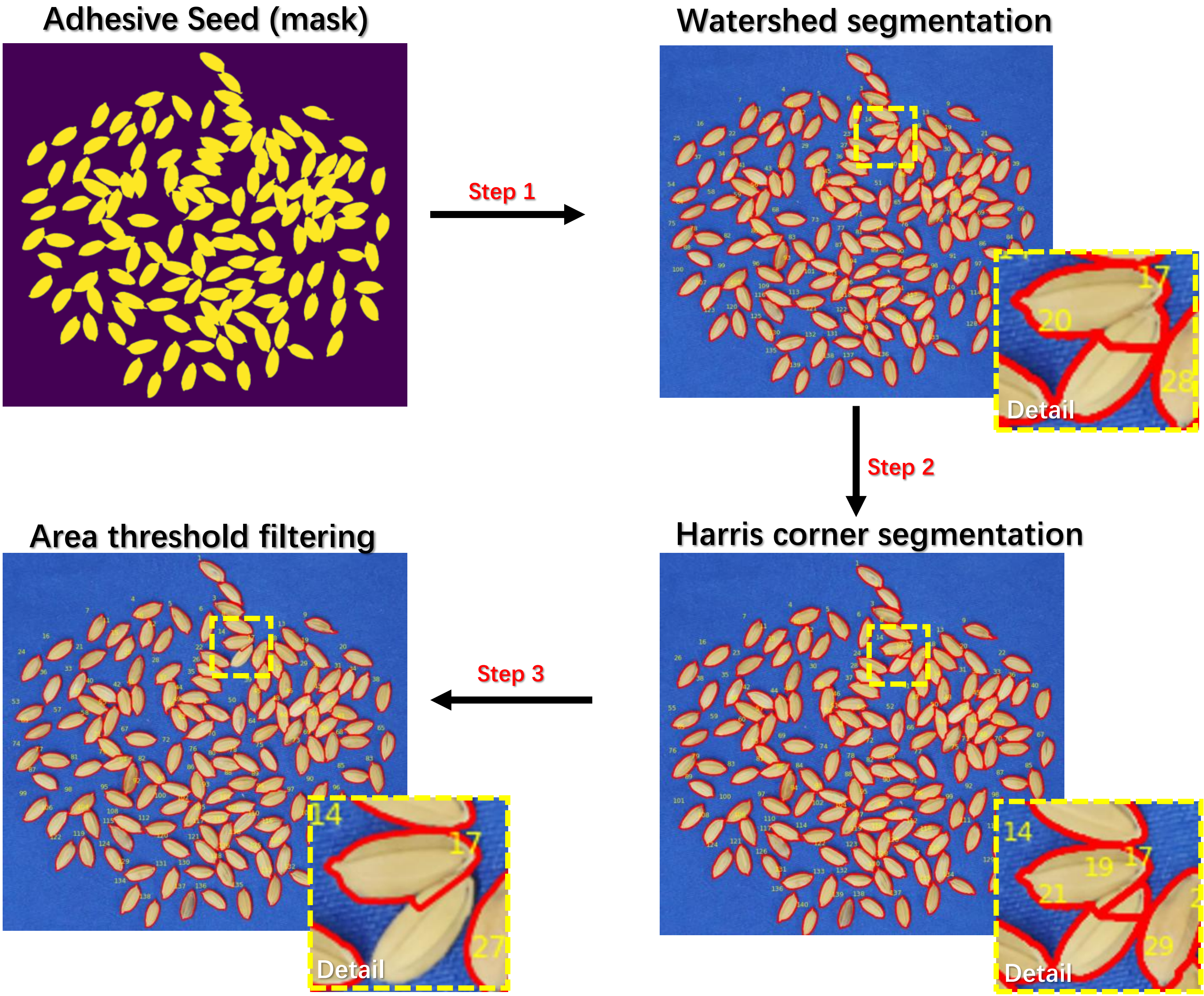

Fig. S7. Image processing pipeline for dense and overlapping seeds

Table S1. Performance comparison of four models on the test dataset (*n*=884).

Model	Accuracy	Precision	Recall	F1-Score
**KNN**	0.992	0.966	1	0.982
**SVM**	0.989	0.951	1	0.975
**RF**	0.992	0.966	1	0.982
**XGBoost**	0.993	0.970	1	0.985

n represent the size of the test set

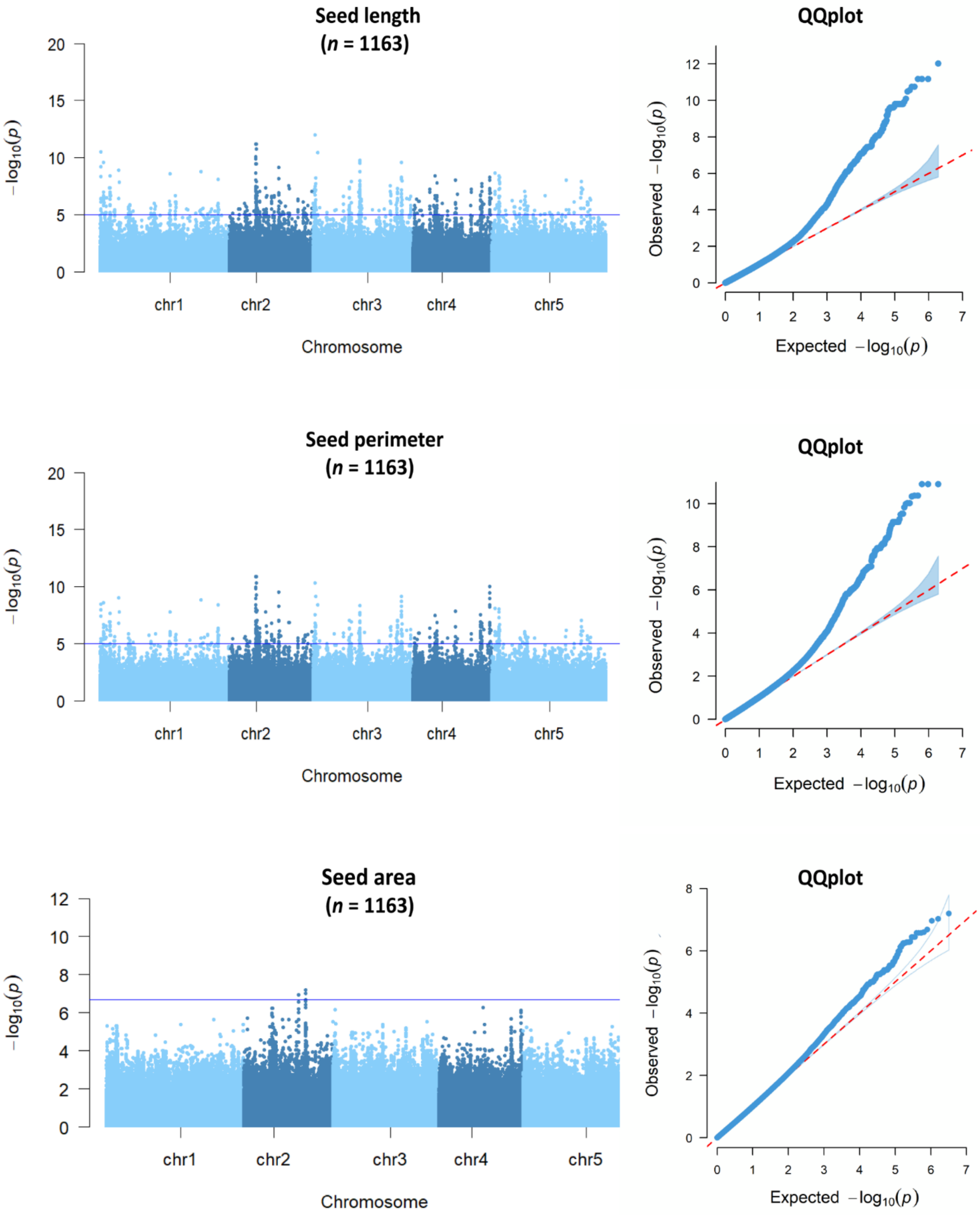
Seed length
(n = 1163)
−log10(p)
0
5
10
15
20
chr1
chr2
chr3
chr4
chr5
Chromosome
QQplot
Observed −log10(p)
Expected −log10(p)
0
2
4
6
8
10
12
0
1
2
3
4
5
6
7
Seed perimeter
(n = 1163)
−log10(p)
0
5
10
15
20
chr1
chr2
chr3
chr4
chr5
Chromosome
QQplot
Observed −log10(p)
Expected −log10(p)
0
2
4
6
8
10
0
1
2
3
4
5
6
7
Seed area
(n = 1163)
−log10(p)
0
2
4
6
8
10
12
chr1
chr2
chr3
chr4
chr5
Chromosome
QQplot
Observed −log10(p)
Expected −log10(p)
0
2
4
6
8
0
1
2
3
4
5
6
7

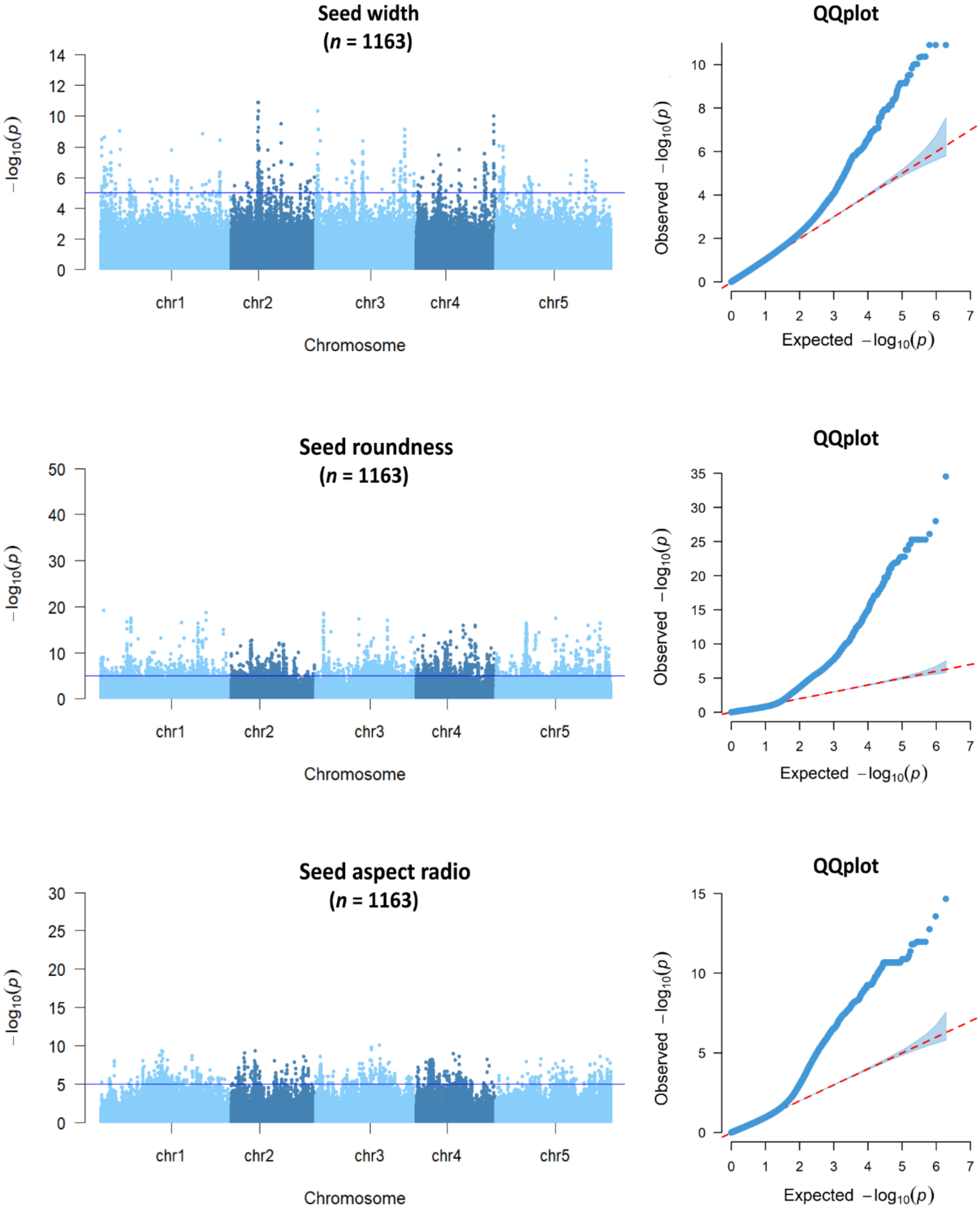
Seed width
(n = 1163)
QQplot
Seed roundness
(n = 1163)
QQplot
Seed aspect radio
(n = 1163)
QQplot
−log10(p)
chr1
chr2
chr3
chr4
chr5
Chromosome
Observed −log10(p)
Expected −log10(p)

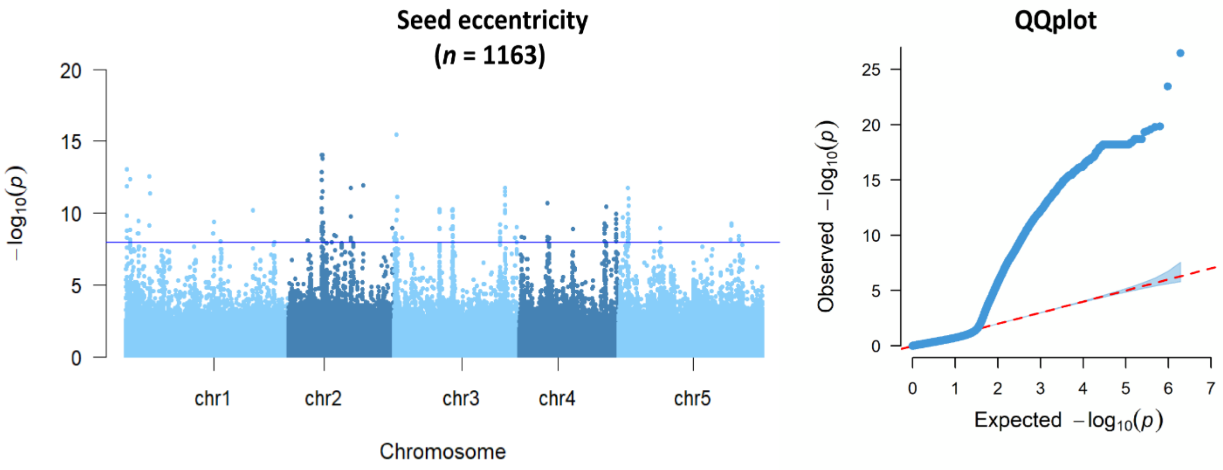

Seed eccentricity
(*n* = 1163)
20
15
10
5
0
$-\log_{10}(p)$
chr1
chr2
chr3
chr4
chr5
Chromosome
QQplot
25
20
15
10
5
0
Observed $-\log_{10}(p)$
0
1
2
3
4
5
6
7
Expected $-\log_{10}(p)$

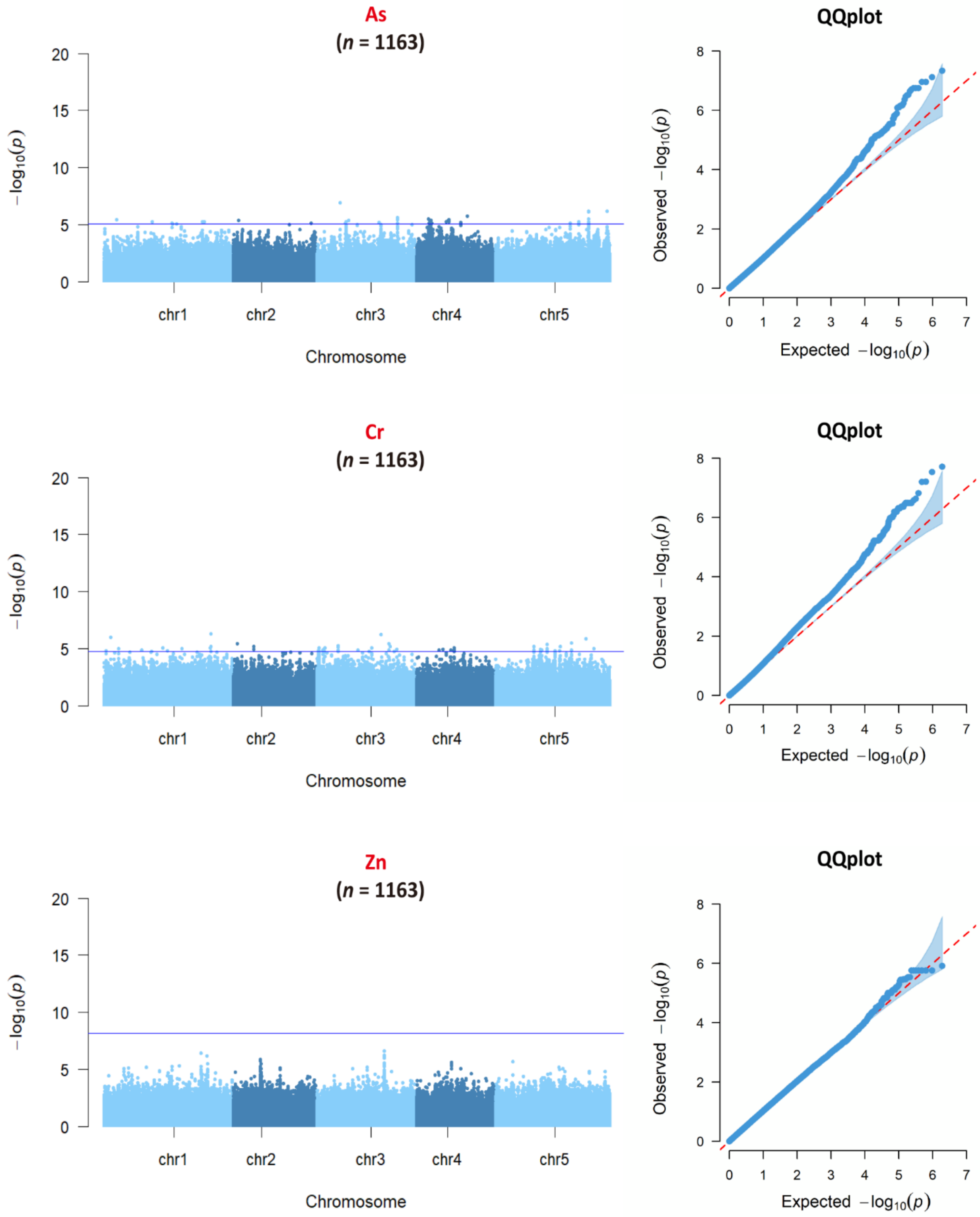

As
(n = 1163)
QQplot
Cr
(n = 1163)
QQplot
Zn
(n = 1163)
QQplot
−log10(p)
Chromosome
chr1
chr2
chr3
chr4
chr5
Observed −log10(p)
Expected −log10(p)

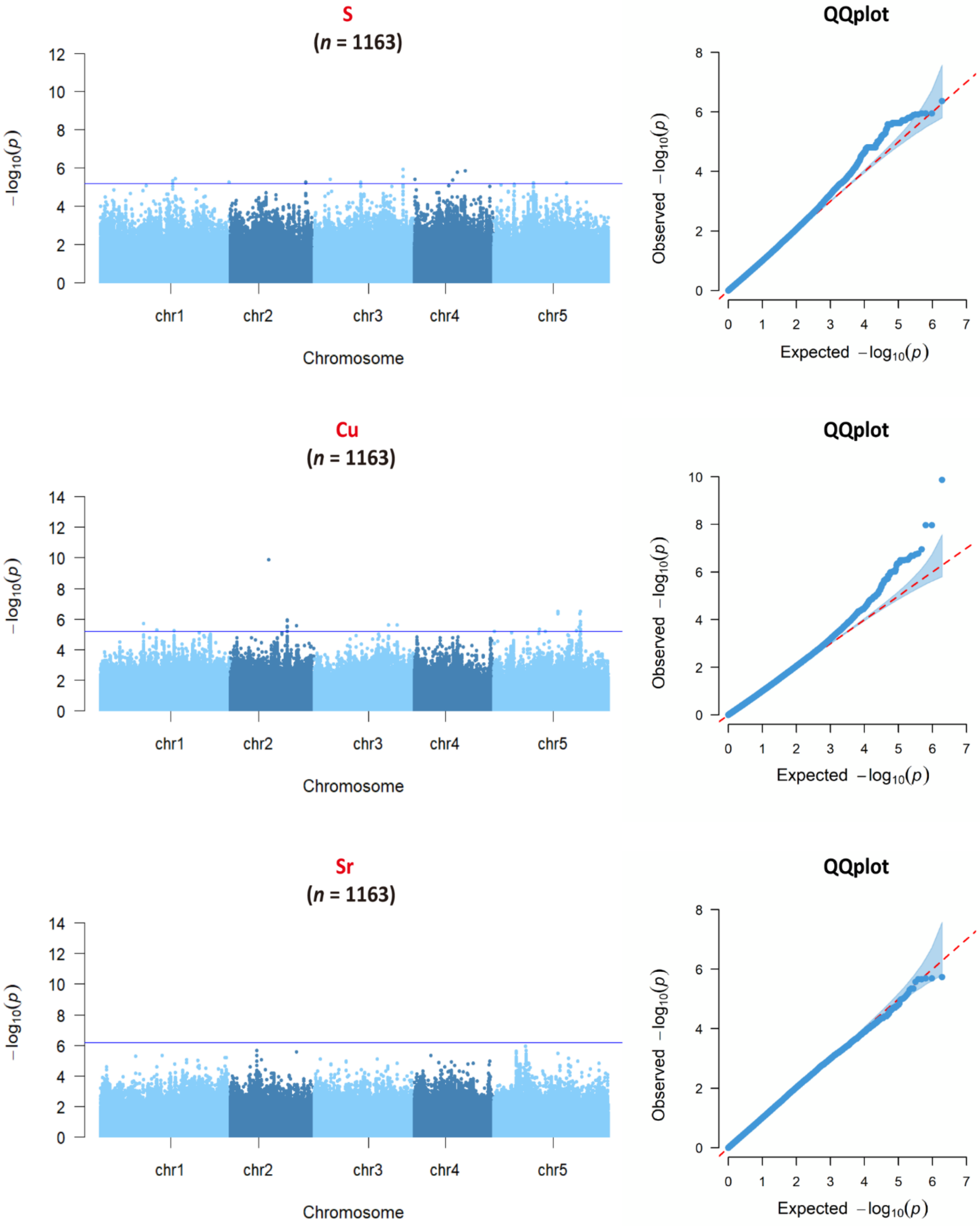
S
(n = 1163)
QQplot
Cu
(n = 1163)
QQplot
Sr
(n = 1163)
QQplot
chr1
chr2
chr3
chr4
chr5
Chromosome
$-\log_{10}(p)$
Observed $-\log_{10}(p)$
Expected $-\log_{10}(p)$

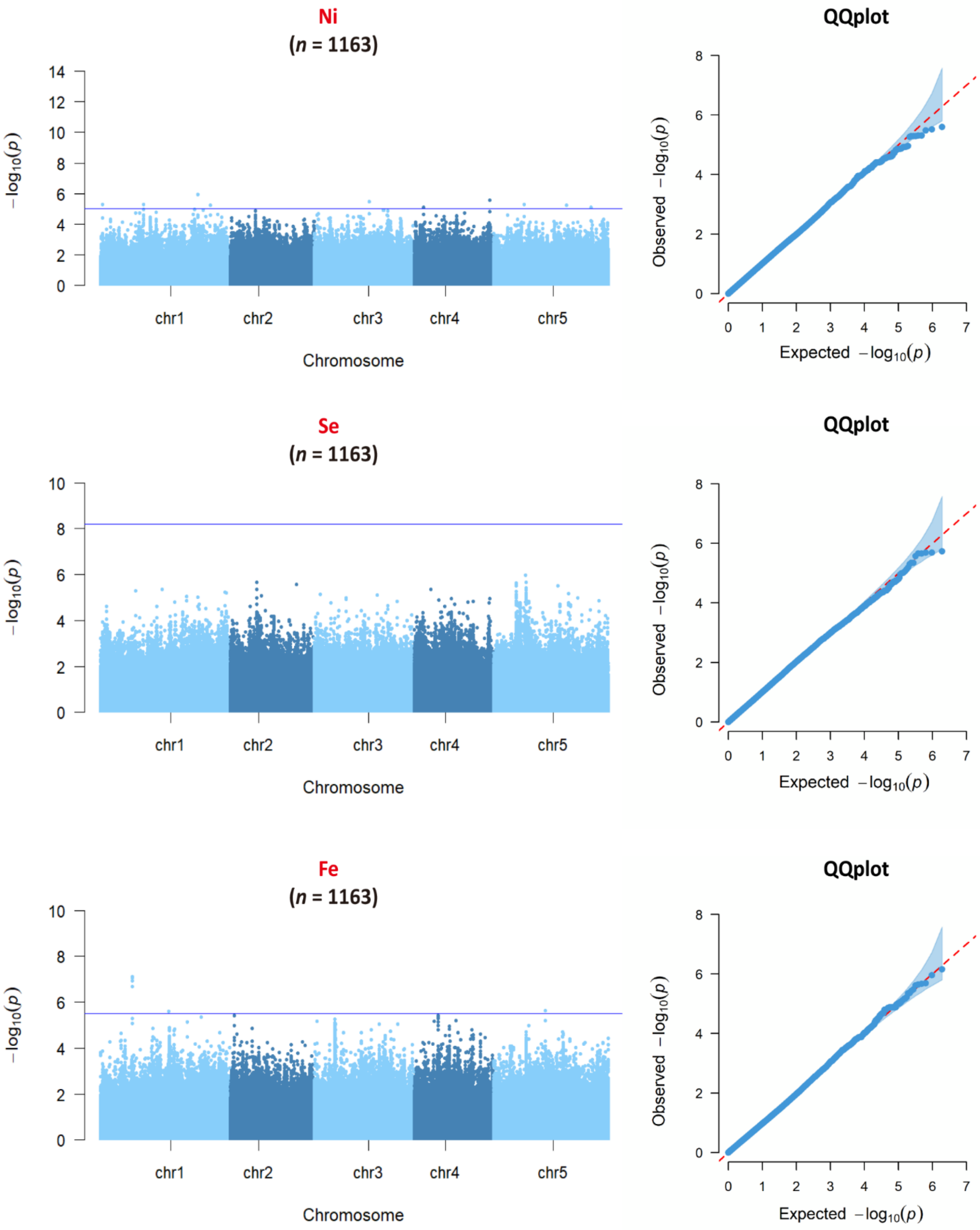

Ni
(n = 1163)
QQplot
−log10(p)
Chromosome
chr1
chr2
chr3
chr4
chr5
Observed −log10(p)
Expected −log10(p)
Se
(n = 1163)
QQplot
−log10(p)
Chromosome
chr1
chr2
chr3
chr4
chr5
Observed −log10(p)
Expected −log10(p)
Fe
(n = 1163)
QQplot
−log10(p)
Chromosome
chr1
chr2
chr3
chr4
chr5
Observed −log10(p)
Expected −log10(p)

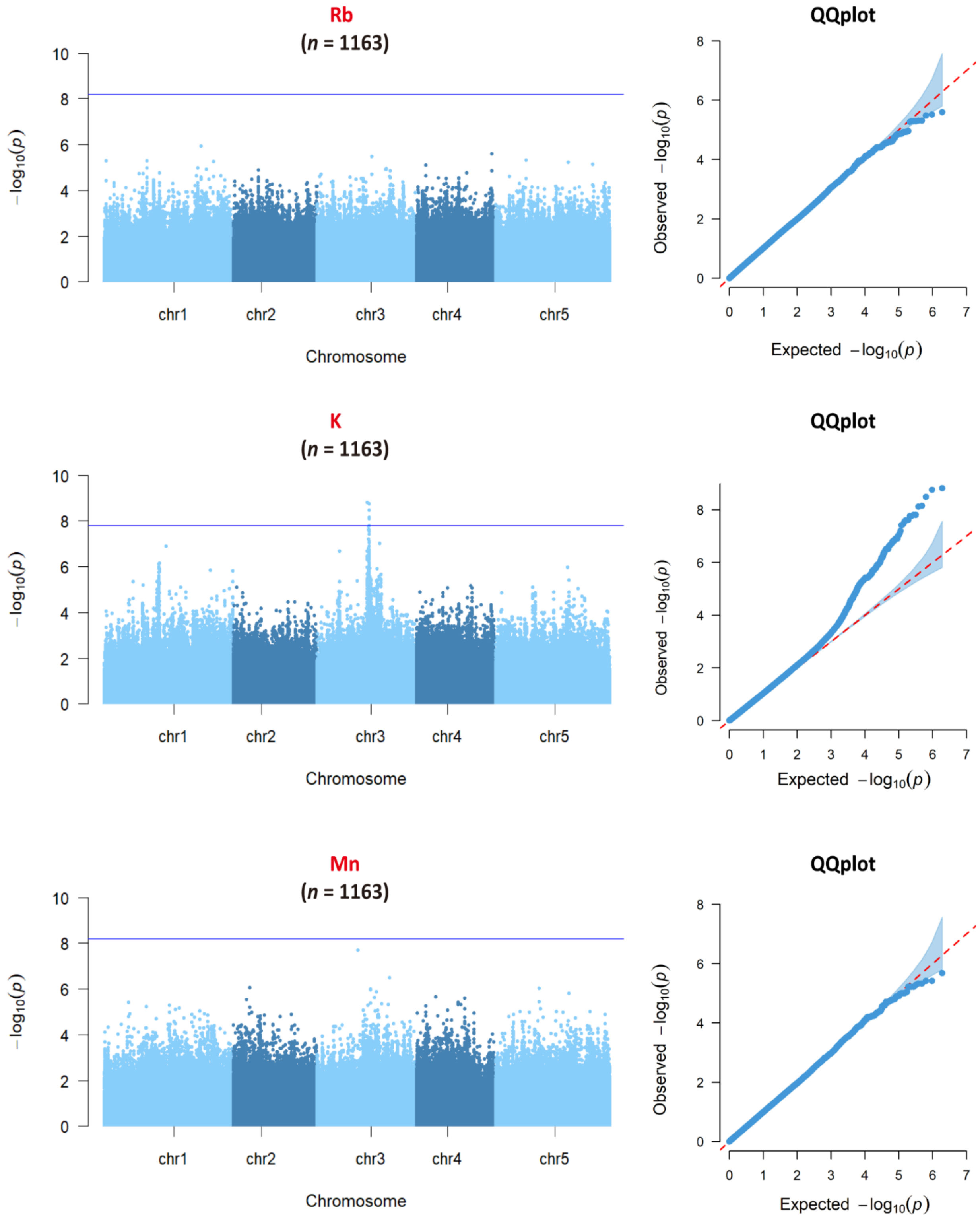
Rb
(n = 1163)
QQplot
K
(n = 1163)
QQplot
Mn
(n = 1163)
QQplot
−log10(p)
chr1
chr2
chr3
chr4
chr5
Chromosome
Observed −log10(p)
Expected −log10(p)

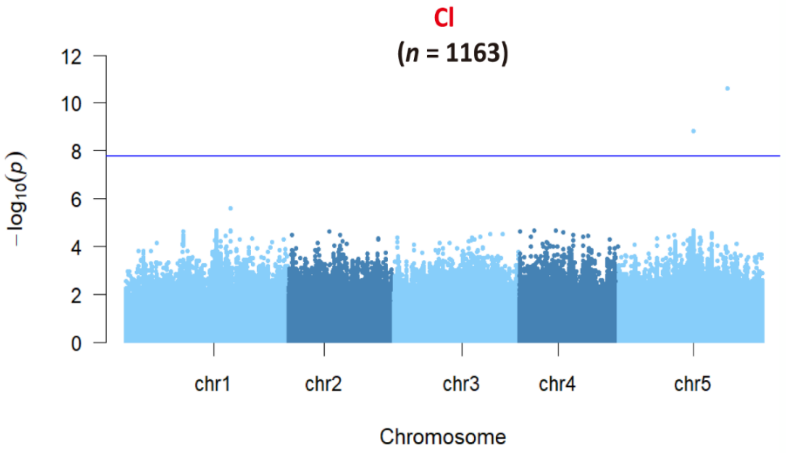
Cl
(n = 1163)
−log10(p)
chr1
chr2
chr3
chr4
chr5
Chromosome

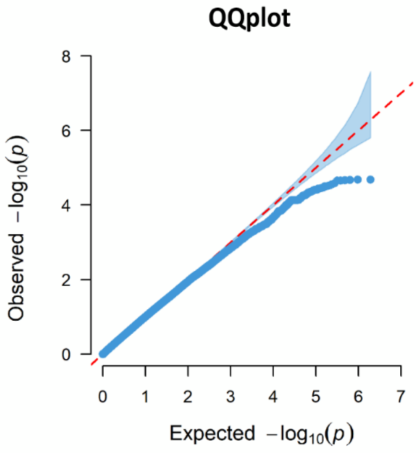
QQplot
Observed −log10(p)
Expected −log10(p)

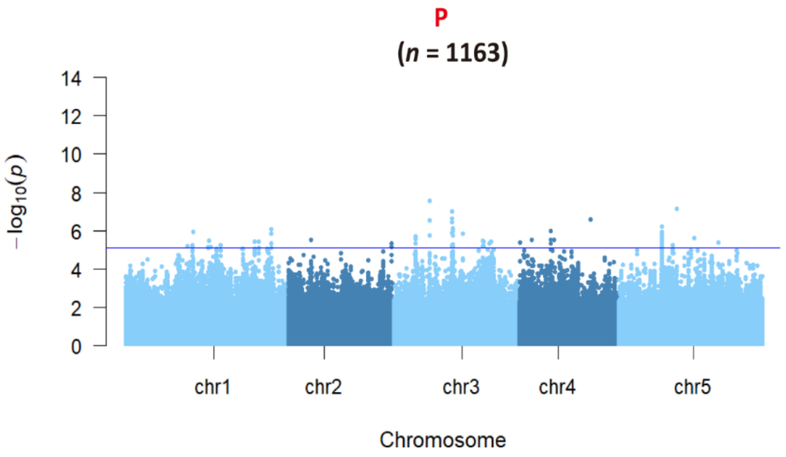
P
(n = 1163)
−log10(p)
chr1
chr2
chr3
chr4
chr5
Chromosome

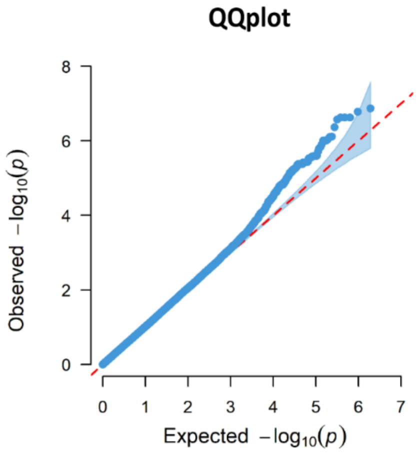
QQplot
Observed −log10(p)
Expected −log10(p)

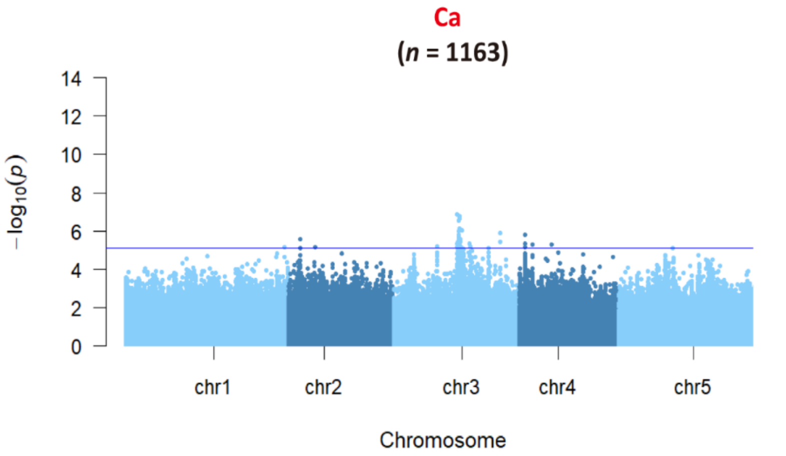
Ca
(n = 1163)
−log10(p)
chr1
chr2
chr3
chr4
chr5
Chromosome

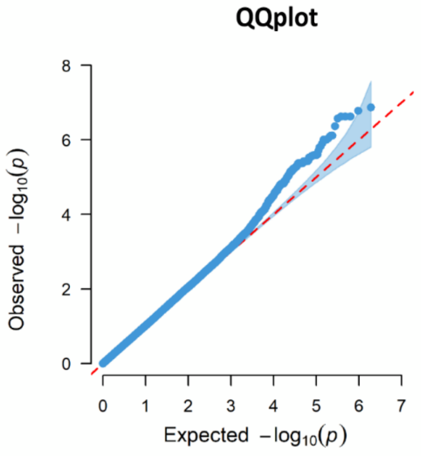
QQplot
Observed −log10(p)
Expected −log10(p)

Fig. S8. Manhattan plot and Q-Q plots based on genome-wide association analysis of significance loci. The blue horizontal lines in the plot represent the significance thresholds, *Benjamini-Hochberg* corrections were applied to determine the threshold for association signals (for details, refer to https://gwas.gmi.oeaw.ac.at/).The red arrows point to the significant loci and we chose *200* kb as the candidate gene interval.
